# Design, Synthesis, and Herbicidal Activity Evaluation of Novel Aryl-Naphthyl Methanone Derivatives

**DOI:** 10.3389/fchem.2019.00002

**Published:** 2019-01-22

**Authors:** Ying Fu, Kui Wang, Peng Wang, Jing-Xin Kang, Shuang Gao, Li-Xia Zhao, Fei Ye

**Affiliations:** Department of Applied Chemistry, College of Science, Northeast Agricultural University, Harbin, China

**Keywords:** 4-hydroxyphenylpyruvate dioxygenase, design, synthesis, aryl-naphthyl methanone, herbicidal activity

## Abstract

4-Hydroxyphenylpyruvate dioxygenase (HPPD) is one of the most vital targets for herbicides discovery. In search for HPPD inhibitors with novel scaffolds, a series of aryl-naphthyl methanone derivatives have been designed and synthesized through alkylation and Friedel-Crafts acylation reactions. The bioassay indicated some of these compounds displayed preferable herbicidal activity at the rate of 0.75 mmol/m^2^ by post-emergence application, in which compound **3h** displayed the best herbicidal activity. The molecular docking showed that compound **3h** could bind well to the active site of the *At*HPPD. This study shows that aryl-naphthyl methanone derivatives could be a potential lead structure for further development of novel herbicides.

## Introduction

4-Hydroxyphenylpyruvate dioxygenase (EC 1.13.11.27, HPPD) is an iron-dependent, nonheme oxygenase present in most organisms. It is involved in the second step of the tyrosine degradation pathway, and catalyses the conversion of 4-hydroxyphenyl pyruvic acid (HPPA) into homogentisic acid (HGA) (Xu et al., 2014; Wang et al., 2015b). HGA is the intermediate of tyrosine catabolism and the precursor of the plastoquinone and tocopherols. The plastoquinone plays an important role in assisting phytoene desaturase to catalyse and synthesize the carotenoid. HPPD inhibiting-based herbicides cause low level of the carotenoid, which indirectly affects the photosynthesis. When exposed to sunlight, plants will develop into albinism followed by gangrene and death treated with HPPD inhibitors (Mitchell et al., 2001; Moran, 2005; Wojcik et al., 2014). There are many advantages of HPPD inhibitors, such as excellent crop selectivity, low application rate, low toxicity, broad-spectrum weed control, and benign environmental effects (Beaudegnies et al., 2009; Elmore et al., 2011; Laschi et al., 2016; Wang et al., 2016).

Up to now, approximately 15 HPPD inhibitors have been commercialized, which are primarily assorted into three classifications: triketones, pyrazoles, and isoxazoles (Zhu et al., 2005; Witschel, 2009; Wang et al., 2015b). Among the commercial HPPD herbicides, pyrazole and isoxazole derivatives have been studied diffusely due to their structural diversity. The first commercial pyrazole product, pyrazolynate, was launched by Sankyo in 1980 for controlling annual and perennial weeds in rice at a dose of 3–4 kg ai/ha (Kubo et al., 2012). Five years later, Ishihara Sangyo Kaisha commercialized the second pyrazole herbicide, pyrazoxyfen, which was applied in irrigated fields for broad-leaved weeds, was used at rates of 3 kg ai/ha (He et al., 2017). In 1990, the isoxaflutole was registered by BASF, using in corn field for selective pre-emergence herbicide at a dose of 120–150 g ai/ha (Luscombe and Pallett, 1996). Nearly, the ultra-efficient herbicide, topramezone, was commercialized by BASF as a postemergence herbicide with excellent selectivity to control all major grasses and broadleaf weeds in corn at a dose of 12–75 g ai/ha (Rene et al., 2003).

**Figure 1:**
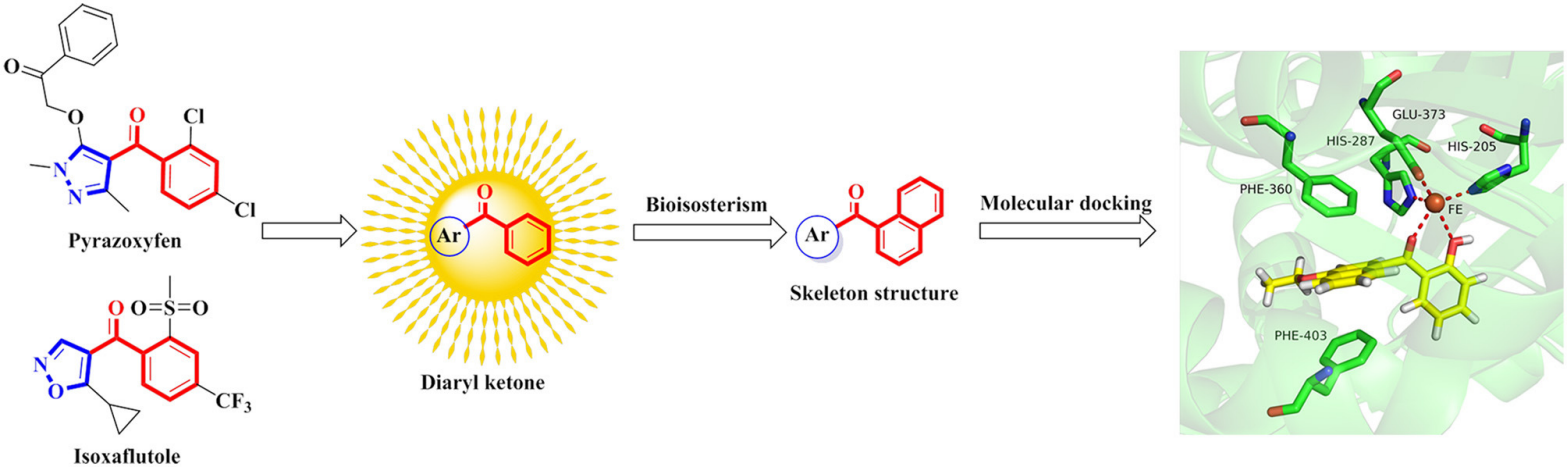
The design strategy of title compounds.

The commercialized herbicide pyrazoxyfen and isoxaflutole were common HPPD inhibitors, and it was found that the active substructure was mainly based on diaryl ketone (Figure 1). Many studies on HPPD inhibitors have proven that improved conjugated system of diaryl ketone will increase the π-π interaction, and enhance the chelation with Fe^2+^ to improve herbicidal activity (Lee et al., 1997; Ahrens et al., 2013; Santucci et al., 2017). For example, the HPPD inhibitor triketone-quinoline exhibited promising and broad-spectrum herbicidal activity at the rate of 150 g ai/ha with increased conjugated system by introducing quinoline group (Wang et al., 2015a). Furthermore, a series of pyrazole-quinoline compounds were included in the BASF patent, which exhibited well weed-inhibiting activity at the dosage of 63–125 g ai/ha (He et al., 2017). As a part of our job in designing and synthesizing novel HPPD inhibitors (Fu et al., 2017a,b, 2018), herein, the skeleton structure was obtained by replacing benzene with naphthalene based on bioisosterism. And the aromatic part was modified with different aromatic heterocycles. Through this strategy, we hoped that the better herbicidal activity would be attained. Twenty-three aryl-naphthyl methanone derivatives were synthesized through alkylation and Friedel-Crafts acylation reactions. The greenhouse experiments showed that compound **3h** at a dose of 2.1 kg ai/ha has better herbicidal activity than pyrazoxyfen at rates of 3 kg ai/ha, and most of the synthesized compounds showed “good” to “excellent” herbicidal activity against barnyard grass at rates of 2–4 kg ai/ha.

## Materials and Methods

### Chemicals and Instruments

All the solvents and reactants were analytical pure and applied without purification. Analytical thin-layer chromatography (TLC) was exercised in silica gel GF_254_. Column chromatographic purification was carried out using silica gel. Yields were not optimized. The melting point was gauged on a Beijing Taike point instrument (X-4) and was uncorrected. The infrared (IR) spectra were collected on a Bruker ALPHA-T spectrometer (in KBr pallets). The nuclear magnetic resonance (NMR) spectrum were collected on a Bruker AV-400 spectrometer using CDCl_3_ as solvent and tetramethylsilane (TMS) as internal standard. The high-resolution mass spectrum was collected on a high resolution mass spectrometer (HRMS) of Bruker. Crystallographic data of the compound was measured on a Rigaku R-AXIS RAPID area-detector diffractometer.

#### General Procedure for Preparation of Intermediate Naphthyl Ether (2a and 2b) (Momeni, 2007)

The naphthol **1a** or **1b** (2 mmol) and anhydrous K_2_CO_3_ (14 mmol) were ground together into fine powder and stirred vigorously at 60°C in 100 mL flask. Alkylating agent (dimethyl sulfate or diethyl sulfate, 3 mmol) was added directly and heated with vigorous stirring for 2 h, and the TLC was used to monitor reaction progress. Water was added to the mixture after the reaction finished, then the solid was filtrated and washed with water. Collect the liquid, the products were extracted with diethyl ether, and the solvent was evaporated. The crude product was purified by column chromatography with petroleum ether and EtOAc (*V:V*=10:1) as eluent or by recrystallization in EtOAc-*n*-hexane (in the case of solid ethers). The spectral data for intermediate **2a** and **2b** are available in the Supporting Information.

#### General Procedure for Preparation of Aryl-Naphthyl Methanone 3a-w

**Figure 2:**
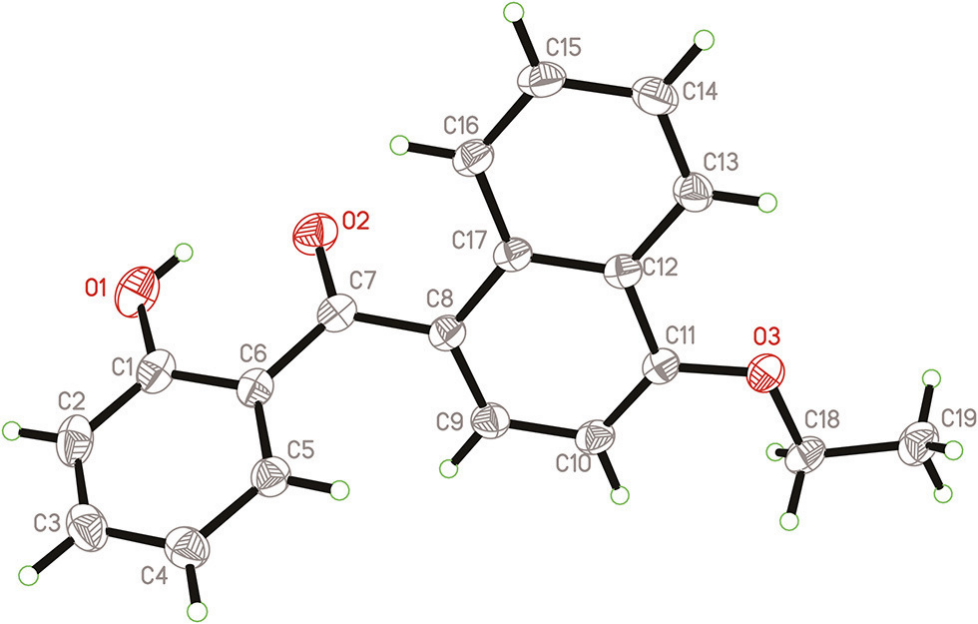
X-ray crystal structure for compound **3h**.

Naphthyl ether (10 mmol) and aroyl chloride (12 mmol) were mixed in dry CH_2_Cl_2_ (50 mL) at 0°C, and adding anhydrous AlCl_3_ (22 mmol) to the solution with stirring. Then the mixture was refluxed and stirred for additional 2 h. After the reaction was completed, the solvent was removed. Then 10% HCl (50 mL) were added to the mixture and stirred vigorously for 30 min, the crude product was obtained after filtration. The crude products were purified by column chromatography with petroleum ether-EtOAc (*V:V* = 15:1) as eluent or by recrystallization in EtOAc-*n*-hexane. In addition, compound **3h** was recrystallized from a mixture of ethyl acetate and petroleum ether to afford a suitable crystal and its structure was determined by single crystal X-ray diffraction analysis. The molecular structure of compound **3h** with atom-numbering is shown in Figure 2.

### Compounds Data

#### (4-Ethoxynaphthalen-1-yl)(Phenyl)Methanone (3a)

White solid; yield, 65%; mp, 109–110°C; IR (KBr, cm^−1^) ν: 3073-2893 (C-H), 1651 (C = O), 1579-1445 (C=C); ^1^H NMR (400 MHz, CDCl_3_, ppm) δ: 8.37-8.47 (d, *J* = 9.8 Hz, 2H, Ar-H), 7.89-7.84 (d, *J* = 7.0 Hz, 2H, Ar-H), 7.63-7.45 (m, 6H, Ar-H), 6.84-6.74 (d, *J* = 8.1 Hz, 1H, Ar-H), 4.26-4.33 (m, 2H, O-CH_2_), 1.59-1.64 (t, *J* = 9.2 Hz, 3H, -CH_3_); ^13^C NMR (100 MHz, CDCl_3_, ppm) δ: 197.36, 157.75, 139.53, 132.71, 132.46, 131.63, 131.63, 130.30, 130.30, 128.26, 128.01, 128.01, 127.83, 125.86, 125.73, 122.38, 102.62, 64.11, 14.74. HRMS (ESI): *m/z* [M+H^+^] calculated for monoisotopic mass 277.1150, found 277.1225.

#### (2-Chlorophenyl)(4-ethoxynaphthalen-1-yl)methanone (3b)

Yellow solid; yield, 59%; mp, 143–144°C; IR (KBr, cm^−1^) ν: 3056-2894 (C-H), 1643 (C=O), 1578-1429 (C=C); ^1^H NMR (400 MHz, CDCl_3_, ppm) δ: 8.55-8.39 (m, 2H, Ar-H), 8.10-7.83 (m, 4H, Ar-H), 7.61-7.64 (m, 3H, Ar-H), 6.79-6.81 (d, *J* = 8.1 Hz, 1H, Ar-H), 4.27-4.34 (m, 2H, O-CH_2_), 1.59-1.64 (t, *J* = 9.2 Hz, 3H, -CH_3_); ^13^C NMR (100 MHz, CDCl_3_, ppm) δ: 196.03, 158.00, 138.91, 137.90, 132.64, 131.68, 131.68, 131.57, 128.62, 128.62, 128.16, 127.46, 125.93, 125.88, 125.63, 122.48, 102.66, 64.20, 14.75. HRMS (ESI): *m/z* [M+H^+^] calculated for monoisotopic mass 311.0761, found 311.0836.

#### (4-Chlorophenyl)(4-ethoxynaphthalen-1-yl)methanone (3c)

White solid; yield, 53%; mp, 138–140°C; IR (KBr, cm^−1^) ν: 3055-2893 (C-H), 1643 (C = O), 1578-1429 (C=C); ^1^H NMR (400 MHz, CDCl_3_, ppm) δ: 8.35-8.43 (m, 2H, Ar-H), 7.79-7.81 (d, *J* = 8.1 Hz, 2H, Ar-H), 7.44-7.60 (m, 5H, Ar-H), 6.79-6.81 (d, *J* = 8.1 Hz, 1H, Ar-H), 4.28-4.35 (m, 2H, O-CH_2_), 1.60-1.64 (t, *J* = 9.2 Hz, 3H, -CH_3_); ^13^C NMR (100 MHz, CDCl_3_, ppm) δ: 195.85, 158.34, 147.21, 141.20, 132.67, 132.56, 130.20, 130.20, 128.41, 128.41, 126.90, 125.97, 125.93, 125.62, 125.55, 122.49, 102.57, 64.23, 14.71. HRMS (ESI): *m/z* [M+H^+^] calculated for monoisotopic mass 311.0761, found 311.0836.

#### (4-Ethoxynaphthalen-1-yl)(4-fluorophenyl)methanone (3d)

Yellow solid; yield, 57%; mp, 117–119°C; IR (KBr, cm^−1^) ν: 3076-2893 (C-H), 1643(C=O), 1578-1430 (C = C); ^1^H NMR (400 MHz, CDCl_3_, ppm) δ: 7.86-8.43 (m, 4H, Ar-H), 7.56-7.59 (m, 3H, Ar-H), 7.09-7.17 (m, 2H, Ar-H), 6.78-6.80 (d, *J* = 8.1 Hz, 1H, Ar-H), 4.25-4.32 (m, 2H, O-CH_2_), 1.59-1.63 (t, *J* = 9.2 Hz, 3H, -C-CH_3_); ^13^C NMR (100 MHz, CDCl_3_, ppm) δ: 199.55, 158.77, 140.99, 137.06, 134.84, 132.73, 130.98, 130.13, 129.12, 128.75, 127.53, 126.01, 125.92, 125.87, 125.32, 122,43, 102.74, 64.19, 14.70. HRMS (ESI): *m/z* [M+H^+^] calculated for monoisotopic mass 295.1056, found 295.1126.

#### (4-Ethoxynaphthalen-1-yl)(4-trifluoromethylphenyl)methanone (3e)

White solid; yield, 56%; mp, 155–156°C; IR (KBr, cm-1) ν: 3053-2887 (C-H), 1634 (C=O), 1572-1429 (C = C); ^1^H NMR (400 MHz, CDCl_3_, ppm) δ: 7.43-8.43 (m, 9H, Ar-H), 6.75-6.80 (d, *J* = 8.1 Hz, 1H, Ar-H), 4.28-4.35 (m, 2H, O-CH_2_), 1.60-1.64 (t, *J* = 9.2 Hz, 3H, -C-CH_3_); ^13^C NMR (100 MHz, CDCl_3_, ppm) δ: 195.85, 158.34, 147.21, 141.20, 132.67, 132.56, 132.56, 130.39, 130.20, 128.41, 128.41, 126.90, 125.97, 125.93, 125.62, 125.55, 122.49, 102.57, 64.23, 14.71. HRMS (ESI): *m/z* [M+H^+^] calculated for monoisotopic mass 345.1024, found 345.1094.

#### (4-Ethoxynaphthalen-1-yl)(4-nitrophenyl)methanone (3f)

Yellow solid; yield, 61%; mp, 152–153°C; IR (KBr, cm^−1^) ν: 3053-2887 (C-H), 1634 (C=O), 1572-1429 (C=C); ^1^H NMR (400 MHz, CDCl_3_, ppm) δ: 8.31-8.57 (m, 4H, Ar-H), 7.95-7.98 (m, 2H, Ar-H), 7.56-7.67 (m, 3H, Ar-H), 6.78-6.81 (d, *J* = 8.2 Hz, 1H, Ar-H), 4.28-4.35 (m, 2H, O-CH_2_), 1.60-1.65 (t, *J* = 9.2 Hz, 3H, -C-CH_3_); ^13^C NMR (100 MHz, CDCl_3_, ppm) δ: 195.12, 158.88, 149.75, 145.08, 133.40, 132.64, 132.64, 130.91, 128.73, 128.73, 126.16, 126.12, 125.95, 125.54, 123.47, 122.60, 102.59, 64.35, 14.69. HRMS (ESI): *m/z* [M+H^+^] calculated for monoisotopic mass 322.1001, found 322.1070.

#### (4-Ethoxynaphthalen-1-yl)(2-methylphenyl)methanone (3g)

Yellow solid; yield, 50%; mp, 126–127°C; IR (KBr, cm^−1^) ν: 3067-2939 (C-H), 1639 (C=O), 1573-1429 (C=C); ^1^H NMR (400 MHz, CDCl_3_, ppm) δ: 8.99-9.02 (d, *J* = 8.5Hz, 1H, Ar-H), 8.42-8.45 (m, 1H, Ar-H), 7.25-7.69 (m, 7H, Ar-H), 6.78-6.71 (d, *J* = 8.2 Hz, 1H, Ar-H), 4.22-4.29 (m, 2H, O-CH_2_), 2.41 (s, 3H, -CH_3_), 1.57-1.62 (t, *J* = 9.2 Hz, 3H, -C-CH_3_); ^13^C NMR (100 MHz, CDCl_3_, ppm) δ: 199.55, 158.77, 140.99, 137.06, 134.84, 132.73, 130.98, 130.13, 129.12, 128.75, 127.53, 126.01, 125.92, 125.87, 125.32, 122,43, 102.74, 64.19, 20.20, 14.70. HRMS (ESI): *m/z* [M+H^+^] calculated for monoisotopic mass 291.1307, found 291.1375.

#### (4-Ethoxynaphthalen-1-yl)(2-hydroxyphenyl)methanone (3h)

Yellow solid; yield, 61%; mp, 125–127°C; IR (KBr, cm^−1^) ν: 3272 (-OH), 3051-2932 (C-H), 1624 (C = O), 1575-1443 (C = C); ^1^H NMR (400 MHz, CDCl_3_, ppm) δ: 12.43 (s, 1H, -OH), 7.99-8.54 (m, 2H, Ar-H), 7.47-7.57 (m, 5H, Ar-H), 6.79-7.13 (m, 3H, Ar-H), 4.28-4.34 (m, 2H, O-CH_2_), 1.61-1.65 (t, *J* = 6.8Hz, 3H, -C-CH_3_); ^13^C NMR (100 MHz, CDCl_3_, ppm) δ: 202.94, 163.42, 157.34, 136.43, 133.95, 132.03, 129.40, 127.87, 127.53, 125.85, 125.81, 125.23, 122.57, 120.78, 118.65, 118.31, 102.81, 64.15, 14.78. HRMS (ESI): *m/z* [M+H^+^] calculated for monoisotopic mass 293.1099, found 293.1175.

#### (4-Ethoxynaphthalen-1-yl)(2-ethoxyphenyl)methanone (3i)

Yellow solid; yield, 32%; mp, 146–147°C; IR (KBr, cm^−1^) ν: 3079-2865 (C-H), 1617 (C=O), 1583-1439 (C = C); ^1^H NMR (400 MHz, CDCl_3_, ppm) δ: 8.81-8.93 (d, *J* = 8.5 Hz, 1H, Ar-H), 8.30-8.43 (d, *J* = 8.5 Hz, 1H, Ar-H), 7.36-7.72 (m, 5H, Ar-H), 6.60-7.10 (m, 3H, Ar-H), 4.22-4.27 (m, 2H, O-CH_2_), 3.85-3.91 (m, 2H, O-CH_2_), 1.55-1.59 (t, *J* = 6.8Hz, 3H, -CH_3_), 0.92-0.96 (t, *J* = 6.8Hz, 3H, -CH_3_); ^13^C NMR (100 MHz, CDCl_3_, ppm) δ: 197.31, 158.27, 157.13, 133.51, 132.48, 131.87, 131.35, 130.09, 128.64, 128.34, 125.94, 125.69, 125.56, 122.17, 120.42, 112.88, 102.73, 64.18, 64.08, 14.71, 14.34. HRMS (ESI): *m/z* [M+H^+^] calculated for monoisotopic mass 321.1412, found 321.1490.

#### (2-Hydroxyphenyl)(4-ethoxynaphthalen-1-yl)methanone (3j)

Yellow solid; yield, 69%; mp, 108–110°C; IR (KBr, cm^−1^) ν: 3032-2917 (C-H), 1606(C=O), 1567-1431 (C=C); ^1^H NMR (400 MHz, CDCl_3_, ppm) δ: 12.41 (s, 1H, -OH), 8.35-8.41 (m, 1H, Ar-H), 8.03-8.09 (m, 1H, Ar-H), 7.45-7.59 (m, 5H, Ar-H), 7.10-7.14 (d, *J* = 0.8 Hz, 1H, Ar-H), 6.78-6.90 (m, 2H, Ar-H), 4.11 (s, 3H, O-CH_3_); ^13^C NMR (100 MHz, CDCl_3_, ppm) δ: 202.94, 163.42, 157.93, 136.47, 133.93, 131.97, 129.20, 127.91, 127.82, 125.97, 125.72, 125.26, 122.44, 120.75, 118.66, 118.32, 102.17, 55.85. HRMS (ESI): *m/z* [M+H^+^] calculated for monoisotopic mass 279.0943, found 279.1018.

#### (4-Methoxynaphthalen-1-yl)(4-methylphenyl)methanone (3k)

White solid; yield, 80%; mp, 122–123°C; IR (KBr, cm^−1^) ν: 3047-2819 (C-H), 1633 (C=O), 1565-1443 (C=C); ^1^H NMR (400 MHz, CDCl_3_, ppm) δ: 8.27-8.43 (m, 2H, Ar-H), 7.73-7.84 (d, *J* = 8.1 Hz, 2H, Ar-H), 7.53-7.62 (m, 3H, Ar-H), 7.25-7.32 (d, *J* = 8.0 Hz, 2H, Ar-H), 6.75-6.87 (d, *J* = 8.0 Hz, 1H, Ar-H), 4.09 (s, 3H, O-CH_3_), 2.46 (s, 3H, CH_3_); ^13^C NMR (100 MHz, CDCl_3_, ppm) δ: 197.15, 158.08, 143.44, 136.70, 132.59, 130.75, 130.54, 130.54, 129.01, 129.01, 128.58, 127.90, 125.80, 125.77, 122.24, 122.24, 102.01, 55.79, 21.72. HRMS (ESI): *m/z* [M+H^+^] calculated for monoisotopic mass 277.1150, found 277.1225.

#### (4-Methoxynaphthalen-1-yl)(4-nitrophenyl)methanone (3l)

Brown solid; yield, 52%; mp, 105–107°C; IR (KBr, cm^−1^) ν: 3060-2923 (C-H), 1629 (C=O), 1561-1431 (C=C); ^1^H NMR (400 MHz, CDCl_3_, ppm) δ: 8.26-8.63 (m, 4H, Ar-H), 7.89-8.03 (d, *J* = 8.8 Hz, 2H, Ar-H), 7.52-7.70 (m, 3H, Ar-H), 6.76-6.87 (d, *J* = 8.2 Hz, 1H, Ar-H), 4.11 (s, 3H, O-CH_3_); ^13^C NMR (100 MHz, CDCl_3_, ppm) δ: 195.12, 159.46, 149.78, 144.96, 133.22, 132.56, 130.93, 130.93, 128.76, 126.42, 126.28, 125.89, 125.56, 123.49, 123.49, 122.50, 102.01, 55.98. HRMS (ESI): *m/z* [M+H^+^] calculated for monoisotopic mass 308.0845, found 308.0919.

#### (4-Trifluoromethylphenyl)(2-methoxynaphthalen-1-yl)methanone(3m)

White solid; yield, 40%; mp, 119–120°C; IR (KBr, cm^−1^) ν: 3040-2820 (C-H), 1660 (C=O), 1566-1423 (C=C); ^1^H NMR (400 MHz, CDCl_3_, ppm) δ: 7.69-8.05 (m, 6H, Ar-H), 7.35-7.56 (m, 4H, Ar-H), 3.84(s, 3H, O-CH_3_); ^13^C NMR (100 MHz, CDCl_3_, ppm) δ: 196.75, 154.43, 148.01, 140.69, 131.89, 131.73, 131.61, 129.79, 129.61, 128.84, 128.30, 127.79, 125.93, 125.69, 125.65, 124.33, 123.74, 112.89, 56.48. HRMS (ESI): *m/z* [M+H^+^] calculated for monoisotopic mass 331.0868, found 331,0943.

#### (2-Methoxynaphthalen-1-yl)(4-nitrophenyl)methanone (3n)

Brown solid; yield, 59%; mp, 125–127°C; IR (KBr, cm^−1^) ν: 3060-2923 (C-H), 1629 (C=O), 1561-1431 (C=C); ^1^H NMR (400 MHz, CDCl_3_, ppm) δ: 8.31-8.57 (m, 4H, Ar-H), 7.95-7.98 (m, 2H, Ar-H), 7.56-7.67 (m, 3H, Ar-H), 6.78-6.81 (d, *J* = 8.2 Hz, 1H, Ar-H), 4.11 (s, 3H, O-CH_3_); ^13^C NMR (100 MHz, CDCl_3_, ppm) δ: 202.94, 163.42, 157.93, 136.47, 133.93, 131.97, 131.97, 129.19, 127.91, 125.97, 125.73, 125.26, 122.44, 120.76, 118.66, 118.32, 102.17, 55.84. HRMS (ESI): *m/z* [M+H^+^] calculated for monoisotopic mass 294.0688, found 294.0759.

#### (4-(1-Methyl-3-trifluoromethyl)pyrazole)(4-ethoxynaphthalen-1-yl)methanone (3o)

White solid; yield, 33%; mp, 109–110°C; IR (KBr, cm^−1^) ν: 3089-2941 (C-H), 1639 (C=O), 1573-1430 (C=C); ^1^H NMR (400 MHz, CDCl_3_, ppm) δ: 8.28-8.68 (m, 2H, Ar-H), 7.45-7.81 (m, 4H, Ar-H), 6.69-6.82 (d, *J* = 8.1 Hz, 1H, Ar-H), 4.25-4.32 (m, 2H, O-CH_2_), 3.97 (s, 3H, CH_3_), 1.58-1.63 (t, *J* = 9.2 Hz, 3H, -C-CH_3_); ^13^C NMR (100 MHz, CDCl_3_, ppm) δ: 188.42, 159.10, 136.79, 132.91, 132.19, 129.28, 129.14, 126.78, 126.65, 126.29, 123.56, 123.16, 119.66, 119.66, 103.28, 64.99, 40.53, 15.47. HRMS (ESI): *m/z* [M+H^+^] calculated for monoisotopic mass 349.1086, found 349.1162.

#### (3-(2,4-Dichlorophenyl)-5-(Trichloromethyl)-1,2,4-Triazole)(4-Ethoxynaphthalen-1-yl)Methanone (3p)

Brown solid; yield, 30%; mp, 166–167°C; IR (KBr, cm^−1^) ν: 3086-2887 (C-H), 1650 (C=O), 1572-1426 (C=C); ^1^H NMR (400 MHz, CDCl_3_, ppm) δ: 8.85-9.04 (d, *J* = 8.4 Hz, 1H, Ar-H), 8.18-8.46 (m, 2H, Ar-H), 7.37-7.77 (m, 5H, Ar-H), 6.78-6.91 (d, *J* = 8.4 Hz, 1H, Ar-H), 4.28-4.35 (m, 2H, O-CH_2_), 1.58-1.62 (t, *J* = 9.2 Hz, 3H, -C-CH_3_); ^13^C NMR (100 MHz, CDCl_3_, ppm) δ: 183.63, 158.99, 158.69, 154.62, 137.42, 135.17, 133.52, 132.99, 132.25, 130.19, 129.90, 128.40, 127.05, 125.34, 125.24, 125.03, 124.14, 121.78, 102.22, 84.78, 63.64, 14.00. HRMS (ESI): *m/z* [M+H^+^] calculated for monoisotopic mass 527.9529, found 527.9607.

#### (4-Ethoxynaphthalen-1-yl)(5-methyl-3-phenyl-4-isoxazole)methanone (3q)

White solid; yield, 71%; mp, 132–133°C; IR (KBr, cm^−1^) ν: 2987-2900 (C-H), 1638 (C=O), 1573-1443 (C=C); ^1^H NMR (400 MHz, CDCl_3_, ppm) δ: 8.13-8.96 (m, 2H, Ar-H), 7.12-7.74 (m, 8H, Ar-H), 6.48-6.67 (d, *J* = 8.2 Hz, 1H, Ar-H), 4.20-4.25 (m, 2H, O-CH_2_), 2.47 (s, 3H, CH_3_), 1.55-1.59 (t, *J* = 6.8 Hz, 3H, -C-CH_3_); ^13^C NMR (100 MHz, CDCl_3_, ppm) δ: 190.12, 172.64, 162.29, 159.02, 133.76, 132.21, 129.52, 128.95, 128.44, 128.44, 128.30, 128.30, 127.26, 126.03, 125.77, 125.29, 122.46, 117.58, 102.72, 102.72, 64.24, 14.61, 12.91. HRMS (ESI): *m/z* [M+H^+^] calculated for monoisotopic mass 358.1365, found 358.1443.

#### (5-Methyl-3-(2-fluoro-6-chlorophenyl)-4-isoxazole)(4-ethoxynaphthalen-1-yl)methanone (3r)

White solid; yield, 70%; mp, 179–180°C; IR (KBr, cm^−1^) ν: 3083-2883 (C-H), 1650 (C=O), 1576-1431 (C=C); ^1^H NMR (400 MHz, CDCl_3_, ppm) δ: 8.11-8.60 (m, 2H, Ar-H), 7.41-7.75 (m, 3H, Ar-H), 6.51-7.21 (m, 4H, Ar-H), 4.18-4.25 (m, 2H, O-CH_2_), 2.57 (s, 3H, CH_3_), 1.54-1.59 (t, *J* = 9.2 Hz, 3H, -C-CH_3_); ^13^C NMR (100 MHz, CDCl_3_, ppm) δ: 188.74, 173.34, 157.99, 155.20, 134.45, 134.40, 131.46, 131.33, 130.90, 130.78, 128.15, 127.05, 125.55, 125.27, 124.87, 121.91, 118.64, 113.79, 113.50, 102.13, 63.85, 14.32, 12.91. HRMS (ESI): *m/z* [M+H^+^] calculated for monoisotopic mass 410.0881, found 410.0961.

#### (5-Methyl-3-(2-fluoro-6-chlorophenyl)-4-isoxazole)(4-ethoxynaphthalen-1-yl)methanone (3s)

White solid; yield, 84%; mp, 140–142°C; IR (KBr, cm^−1^) ν: 3055-2821 (C-H), 1638 (C=O), 1562-1431 (C=C); ^1^H NMR (400 MHz, CDCl_3_, ppm) δ: 8.21-8.50 (m, 2H, Ar-H), 7.47-7.67 (m, 3H, Ar-H), 7.12-7.19 (m, 1H, Ar-H), 7.01-7.06 (d, *J* = 8.1 Hz, 1H, Ar-H), 6.59-6.87 (m, 2H, Ar-H), 4.01 (s, 3H, O-CH_3_), 2.58 (s, 3H, CH_3_); ^13^C NMR (100 MHz, CDCl_3_, ppm) δ: 189.10, 173.71, 161.68, 158.92, 155.53, 131.67, 131.54, 131.25, 131.16, 128.52, 127.59, 126.01, 125.46, 125.20, 125.17, 122.11, 118.91, 114.09, 113.87, 101.79, 55.83, 13.28. HRMS (ESI): *m/z* [M+H^+^] calculated for monoisotopic mass 396.0724, found 396.0800.

#### (4-Ethoxynaphthalen-1-yl)(5-methyl-3-phenyl-4-isoxazole)methanone (3t)

White solid; yield, 86%; mp, 155–156°C; IR (KBr, cm^−1^) ν: 3033-2919 (C-H), 1629 (C=O), 1562-1432 (C=C); ^1^H NMR (400 MHz, CDCl_3_, ppm) δ: 8.75-8.83 (d, *J* = 8.5 Hz, 1H, Ar-H), 8.26-8.35 (m, 1H, Ar-H), 7.45-7.73 (m, 5H, Ar-H), 7.25-7.30 (d, *J* = 4.1 Hz, 1H, Ar-H), 7.17-7.23 (d, *J* = 7.6 Hz, 2H, Ar-H), 6.56-6.66 (d, *J* = 8.2 Hz, 1H, Ar-H), 4.02 (s, 3H, O-CH_3_), 2.47 (s, 3H, CH_3_); ^13^C NMR (100 MHz, CDCl_3_, ppm) δ: 190.15, 172.73, 162.31, 159.61, 133.55, 132.13, 129.53, 129.53, 128.98, 128.44, 128.30, 127.58, 127.58, 126.15, 125.70, 125.31, 122.35, 117.56, 117.56, 102.09, 55.87, 12.93. HRMS (ESI): *m/z* [M+H^+^] calculated for monoisotopic mass 344.1208, found 344.1286.

#### (4-Ethoxynaphthalen-1-yl)(furan-2-yl)methanone (3u)

Yellow solid; yield, 61%; mp, 123–124°C; IR (KBr, cm^−1^) ν: 3126-2940 (C-H), 1631 (C=O), 1577-1428 (C=C); ^1^H NMR (400 MHz, CDCl_3_, ppm) δ: 8.31-8.47 (m, 2H, Ar-H), 7.47-7.89 (m, 4H, Ar-H), 7.02-7.13 (d, *J* = 3.3 Hz, 1H, Ar-H), 6.76-6.86 (d, *J* = 8.1 Hz, 1H, Ar-H), 6.54-6.62 (m, 1H, Ar-H), 4.26-4.33 (m, 2H, O-CH_2_), 1.58-1.63 (t, *J* = 9.2 Hz, 3H, -C-CH_3_); ^13^C NMR (100 MHz, CDCl_3_, ppm) δ: 183.66, 157.91, 153.40, 147.20, 132.29, 130.43, 128.09, 127.14, 125.87, 125.83, 125.28, 122.36, 120.59, 112.09, 102.61, 64.12, 14.74. HRMS (ESI): *m/z* [M+H^+^] calculated for monoisotopic mass 267.0943, found 267.1014.

#### (Furan-2-yl)(4-methoxynaphthalen-1-yl)methanone (3v)

Yellow solid; yield, 65%; mp, 133–134°C; IR (KBr, cm^−1^) ν: 3089-2916 (C-H), 1620 (C=O), 1567-1456 (C=C); ^1^H NMR (400 MHz, CDCl_3_, ppm) δ: 8.31-8.47 (m, 2H, Ar-H), 7.47-7.89 (m, 4H, Ar-H), 7.02-7.13 (d, *J* = 3.3 Hz, 1H, Ar-H), 6.76-6.86 (d, *J* = 8.1 Hz, 1H, Ar-H), 6.54-6.62 (m, 1H, Ar-H), 4.01 (s, 3H, O-CH_3_); ^13^C NMR (100 MHz, CDCl_3_, ppm) δ: 185.12, 157.24, 152.08, 147.65, 137.72, 134.55, 129.75, 129.73, 127.05, 126.32, 124.10, 121.68, 120.72, 112.66, 112.33. HRMS (ESI): *m/z* [M+H^+^] calculated for monoisotopic mass 253.0786, found 253.0861.

#### (Furan-2-yl)(2-hydroxylnaphthalen-1-yl)methanone (3w)

Yellow solid; yield, 43%; mp, 102–103°C; IR (KBr, cm^−1^) ν: 3220 (-OH), 3114-2904 (C-H), 1635 (C = O), 1566-1447 (C = C); ^1^H NMR (400 MHz, CDCl_3_, ppm) δ: 11.14 (s, 1H, -OH), 8.84 (s, 1H, Ar-H), 7.82-7.91 (m, 2H, Ar-H), 7.69-7.75 (d, *J* = 8.4 Hz, 1H, Ar-H), 7.46-7.59 (m, 2H, Ar-H), 7.33-7.41 (m, 2H, Ar-H), 6.68-6.75 (m, 1H, Ar-H); ^13^C NMR (100 MHz, CDCl_3_, ppm) δ: 185.12, 157.24, 152.08, 147.65, 137.72, 134.55, 129.75, 129.73, 127.05, 126.32, 124.10, 121.68, 120.72, 112.66, 112.33. HRMS (ESI): *m/z* [M+H^+^] calculated for monoisotopic mass 253.0786, found 253.1984.

### X-Ray Diffraction Analysis

The cell dimensions and strengths of compound **3h** (0.54 × 0.49 × 0.39 mm) were using a gauged at 298 K on Rigaku R-AXIS RAPID area detector diffractometer with graphite monochromated Mo-*K*α radiation (λ = 0.71073 Å); θ_max_ = 27.48; 13900 measured reflections, 3397 independent reflections (*R*_int_ = 0.0296). The structure was solved by direct method using *SHELXS* 97 and refined with *SHELXL* 97 (Sheldrick, 1997). Full-matrix least-squares refinement based on *F*^2^ using the weight of ω = 1/[σ^2^($F_{\text{o}}^{2}$) + (0.0702*P*)^2^+0.0736*P*] gave final values of *R* = 0.0568, ω*R* = 0.1262, max/min residual electron density = 0.182 and−0.222 e.Å^−3^. Crystallographic data has been deposited at the Cambridge Crystallographic Data Center as supplementary publication number CCDC 1856302. Copies of the data can be obtained, free of charge, on application to CCDC, 12 Union Road, Cambridge CB21EZ, UK [www.ccdc.cam.ac.uk/data_request/cif].

As shown in Figure 2, compound **3h** is compose of three rings, and the bond lengths and bond angles in the structure of **3h** are in the usual ranges. It is obviously that there is π-π conjunctive effect between benzene ring, C7=O2 and naphthalene ring, which caused shorter bond length of C6-C7 [1.48(2) Å] and C7-C8 [1.49(2) Å] than the typical C-C bond length [1.54 Å]. The torsion angle of C11-O3-C18-C19 is 175.75(1)°, which indicated the ethoxy and the naphthalene ring are in a coplanar state. The molecule structure is almost at the same plane, due to the large conjugate system and torsion angle.

### Biological Assays

Herbicidal activities were determined using barnyard grass as indicating crops. The barnyard grass was planted in pots and grown in a greenhouse at around 25°C. The spraying treatment was conducted at rates of 2–4 kg ai/ha when the barnyard grass achieved the two-leaf period. After 7d, the chlorophyll content was surveyed and evaluated (Table 1). The commercial herbicide pyrazoxyfen was chosen as a positive control. The content of chlorophyll a (*C*_*a*_), chlorophyll b (*C*_*b*_), and total chlorophyll (*C*_*t*_) were determined according to the literature (Wang et al., 2013).

**Table 1:** The chlorophyll content processed by compounds **3a-w**.

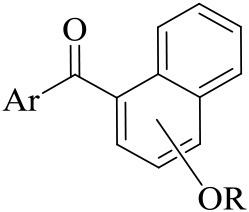					
**Compound**	**OR**	**Ar**	**Barnyard grass**		
			***C**_***a***_* **(mg/g)**	***C**_***b***_* **(mg/g)**	***C**_***t***_***(mg/g)**
CK	–	–	2.032 ± 0.101	1.061 ± 0.093	3.057 ± 0.112
pyrazoxyfen	–	–	0.387 ± 0.063	0.151 ± 0.091	0.537 ± 0.073
**3a**	*p*-OEt	C_6_H_5_	0.974 ± 0.086	0.354 ± 0.109	1.326 ± 0.154
**3b**	*p*-OEt	*o*-Cl-C_6_H_4_	0.827 ± 0.072	0.408 ± 0.117	1.238 ± 0.116
**3c**	*p*-OEt	*p*-Cl-C_6_H_4_	0.863 ± 0.122	0.430 ± 0.109	1.291 ± 0.120
**3d**	*p*-OEt	*p*-F-C_6_H_4_	0.816 ± 0.132	0.358 ± 0.078	1.172 ± 0.129
**3e**	*p*-OEt	*p*-CF_3_-C_6_H_4_	0.401 ± 0.021	0.172 ± 0.092	0.573 ± 0.112
**3f**	*p*-OEt	*p*-NO_2_-C_6_H_4_	0.612 ± 0.094	0.255 ± 0.108	0.867 ± 0.104
**3g**	*p*-OEt	*o*-CH_3_-C_6_H_4_	1.094 ± 0.019	0.524 ± 0.008	1.616 ± 0.011
**3h**	*p*-OEt	*o*-OH-C_6_H_4_	0.321 ± 0.158	0.116 ± 0.057	0.436 ± 0.116
**3i**	*p*-OEt	*o*-OEt-C_6_H_4_	0.750 ± 0.122	0.686 ± 0.108	1.440 ± 0.111
**3j**	*p*-OMe	*o*-OH-C_6_H_4_	0.655 ± 0.104	0.246 ± 0.098	0.901 ± 0.104
**3k**	*p*-OMe	*p*-CH_3_-C_6_H_4_	0.518 ± 0.104	0.955 ± 0.124	1.471 ± 0.078
**3l**	*p*-OMe	*p*-NO_2_-C_6_H_4_	0.686 ± 0.095	0.356 ± 0.028	1.091 ± 0.103
**3m**	*o*-OMe	*p*-CF_3_-C_6_H_4_	0.435 ± 0.124	0.186 ± 0.107	0.622 ± 0.103
**3n**	*o*-OMe	*p*-NO_2_-C_6_H_4_	0.825 ± 0.083	0.316 ± 0.104	1.139 ± 0.107
**3o**	*p*-OEt	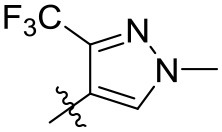	0.498 ± 0.090	0.191 ± 0.102	0.689 ± 0.093
**3p**	*p*-OEt	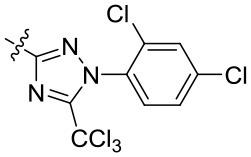	0.591 ± 0.106	0.188 ± 0.100	0.778 ± 0.106
**3q**	*p*-OEt	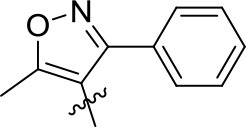	1.486 ± 0.124	0.653 ± 0.021	2.136 ± 0.103
**3r**	*p*-OEt	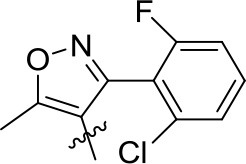	1.895 ± 0.082	0.939 ± 0.057	2.830 ± 0.124
**3s**	*p*-OMe	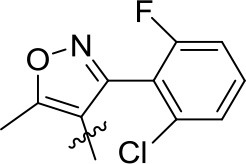	1.562 ± 0.102	0.783 ± 0.055	2.343 ± 0.107
**3t**	*p*-OMe	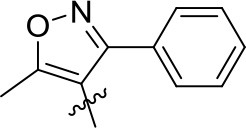	1.564 ± 0.106	0.679 ± 0.045	2.240 ± 0.084
**3u**	*p*-OEt	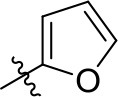	0.405 ± 0.027	0.626 ± 0.121	1.030 ± 0.106
**3v**	*p*-OMe	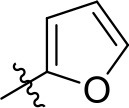	1.248 ± 0.115	0.622 ± 0.071	1.868 ± 0.103
**3w**	*o*-OH	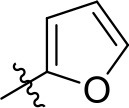	2.018 ± 0.126	0.835 ± 0.091	2.883 ± 0.117

*(0.75 mmol/m^2^). CK is for water treated*.

### Computational Methods

The structure of compounds **3h** and inhibitor DAS869 were constructed by the sketch module of Tripos Associates, Inc. (2001). Subsequently, the Gasteiger-Huckel method was employed to calculate the partial atomic charges and the molecules were optimized. The crystal structure of Arabidopsis thaliana HPPD (*At*HPPD, PDB ID 1TFZ) was obtained from the Protein Data Bank (PDB). Docking was modeled using the CDOCKER method in Accelrys Discovery Studio 2.5 (Catalyst, Version 4.10, 2005). In the preparation of protein structure, water and some other co-crystallized small molecules were removed, and a CHARMM force field was appended. After that, the binding site was limited in a 13.0 Å subset region from the center of the known ligand. The prepared protein and compounds were used to be docking receptor and ligand, respectively. In the process of docking, after the energy minimization using the smart minimize way, the top 10 conformations were saved for every ligand based on -CDOCKER ENERGY, and the remaining parameters were set to the default values.

## Results and Discussion

### Synthetic Chemistry

**Figure 3:**
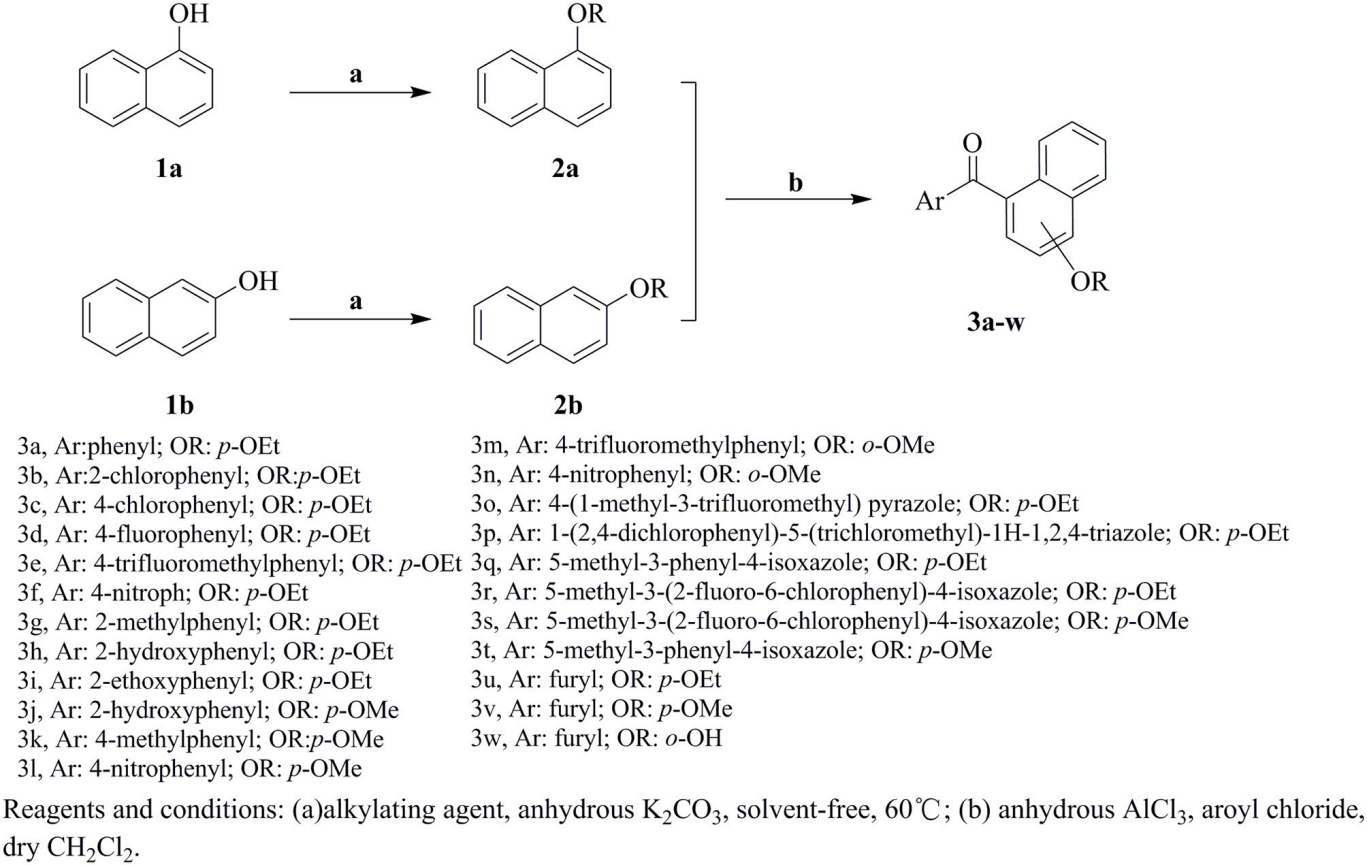
The synthetic route of the compounds **3a-w**.

The synthetic route for the preparation of compounds **3a-w** is depicted in Figure 3. In the process (**a**), the intermediate **2a** was synthesized using naphthol and dimethyl sulfate as the starting materials, and anhydrous K_2_CO_3_ as the acid-binding agent. It should be noted that this process was solvent-free reaction, and the mixing rate of naphthol and the alkylating agents played a vital role. The insufficient mixing rate resulted in low yields. In addition, the effect of acid-binding agents was explored. As shown in Table 2, it was found that anhydrous K_2_CO_3_ was a better acid-binding agent than anhydrous NaHCO_3_ and Et_3_N. To further improve the yield, the ratios of naphthol and the alkylating agents were evaluated. The results indicated that naphthol-alkylating agent ratio in 1:1.5 provided better yields than ratio 1:1 or 1:2.5.

**Table 2:** Explorations of the reaction conditions of intermediate **2a**.

**Entry**	**Acid-binding agent**	**Ratio**	**Yield**
1	K_2_CO_3_	1:1	43%
2	K_2_CO_3_	1:1.5	69%
3	K_2_CO_3_	1:2.5	54%
4	NaHCO_3_	1:1.5	55%
5	Et_3_N	1:1.5	45%

The target compounds **3a-w** were synthesized via Friedel-Crafts acylation reaction. The synthesis of **3a** was first carried out with ZnCl_2_ as catalysts, CHCl_3_ as solvent, at room temperature for 24 h, however, the target compound were not obtained. The different Lewis acids catalysts were evaluated. The results indicated that anhydrous AlCl_3_ was the best catalysts with excellent yield than SnCl_4_, ZnCl_2_, or FeCl_3_. To promote the yields, the solvent influences were investigated as well. The experiment results showed that CH_2_Cl_2_ possessed better yield than CH_3_NO_2_, CS_2_, and CHCl_3_. The detail exploration of the synthetic process to compound **3a** is shown in Table 3.

**Table 3:** Explorations of the reaction conditions of compound **3a**.

**Entry**	**Catalysts**	**Solvent**	**Yield**
1	AlCl_3_	CH_2_Cl_2_	65%
2	AlCl_3_	CH_3_NO_2_	29%
3	AlCl_3_	CS_2_	25%
4	AlCl_3_	CHCl_3_	54%
5	ZnCl_2_	CH_2_Cl_2_	31%
6	FeCl_3_	CH_2_Cl_2_	38%
7	SnCl_4_	CH_2_Cl_2_	34%

The effects of substituent pattern on the yields were studied. It was found that different aromatic substitutents displayed diverse yields comparing compounds **3g**, **3o-q**, and **3u**, and the order was as following: isoxazole>furan>benzene>pyrazole>triazole. The substituted position on the naphthalene ring was also explored. Generally the yield of α-substitution was better than β-substitution, and the yield with methoxy substituted was higher than that of ethoxy, for example, the yield of compound **3e** with 56% was better than compound **3m**. Moreover, the yields of compounds **3q** and **3r** with ethoxy substituted were lower than compounds **3s** and **3t** with methoxy substituted, which was mainly due to the steric hindrance of ethoxy was greater than methoxy.

### Biological Activity and the Structure-Activity Relationships (SAR)

The herbicidal activities of compounds **3a-w** (2–4 kg ai/ha) against barnyard grass in the greenhouse are list in Table 1. The chlorophyll content was gauged after spraying with compounds **3a-w** for 7 days, and the commercial herbicide pyrazoxyfen was selected as positive control. As anticipated, the treated barnyard grass displayed unique bleaching symptoms, as typical HPPD herbicides. The biological activity evaluation indicated that most of the synthetic compounds showed good to excellent herbicidal activity. Compound **3h** at a dose of 2.1 kg ai/ha exhibited better herbicidal activity than pyrazoxyfen at rates of 3 kg ai/ha.

As shown in Table 1, all of the compounds **3a-w** decreased the content of *C*_*a*_, *C*_*b*_, and *C*_*t*_ in different degrees. Comparing the compounds **3a-n**, all of them showed good herbicidal activity, especially compound **3h** exhibited the best activity with a hydroxyl on the *o*-benzene ring, even better than pyrazoxyfen with *o*-PhCOCH_2_O, which may be attributed to the steric effects. It should be noted that the electron-withdrawing groups CF_3_ (**3e, 3m**), NO_2_ (**3f, 3l**, and **3n**), F (**3d**), and Cl (**3b, 3c**) introduced on the phenyl were more beneficial for the herbicidal activity than electron-donating groups CH_3_ (**3g, 3k**) and OEt (**3i**). And it was observed that the stronger electron-withdrawing ability exhibited the better herbicidal activity. For example, compound **3e** showed higher herbicidal activity than compounds **3b** and **3c**. In the same cases, the electron-donating substitution showed weak herbicidal activity. Thus, the SAR could be summarized as follow: CF_3_>NO_2_>F>Cl>H>OEt>CH_3_. The substituted position on the naphthalene ring was also compared. Generally the herbicidal activity of α-substitution was better than β-substitution. For instance, the herbicidal activity of compound **3l** with *p*-OMe was better than compound **3m** with *o*-OMe. Furthermore, the aromatic moiety also affected the inhibition. The results illustrated that benzene ring performed higher herbicidal activity than pyrazole. Compounds **3e** exhibited better activity than compound **3o**. In the same way, as the aromatic ring was triazole (**3p**), it displayed worse herbicidal activity than pyrazole (**3o**), and the isoxazole (**3q**) exhibited lower herbicidal activity than furan (**3u**). For the synthetic compounds, different aromatic subunits displayed diverse herbicidal activity, and the substituted benzene exhibited better herbicidal activity than others aromatic heterocycles.

### Docking Study

**Figure 4:**
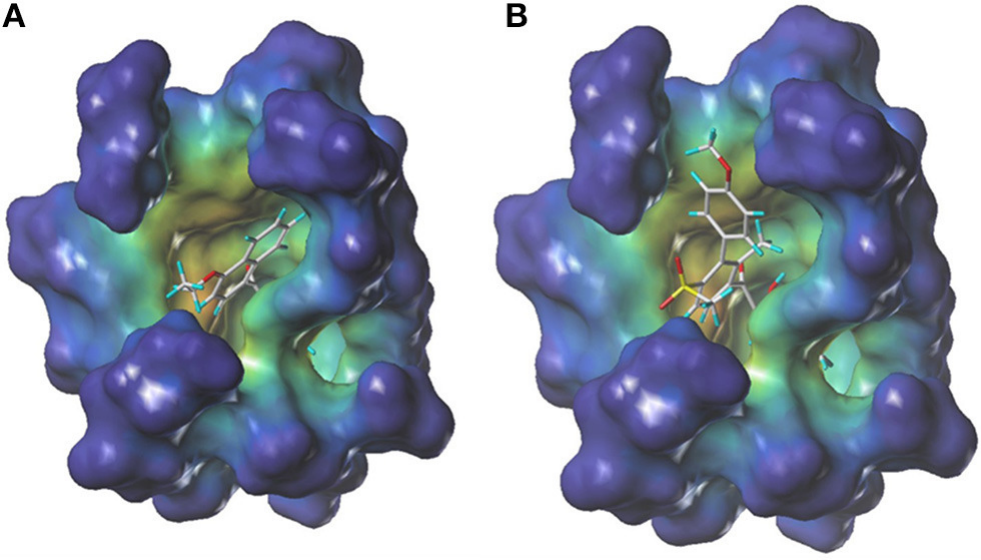
The docking modeling of **3h (A)** and DAS869 **(B)** with *At*HPPD at active site. The carbon atoms are shown in gray, the hydrogen atoms are shown in cyans, the oxygen atoms are shown in red, and the nitrogen atoms are shown in blue, the sulfur atom is shown in yellow.

**Figure 5:**
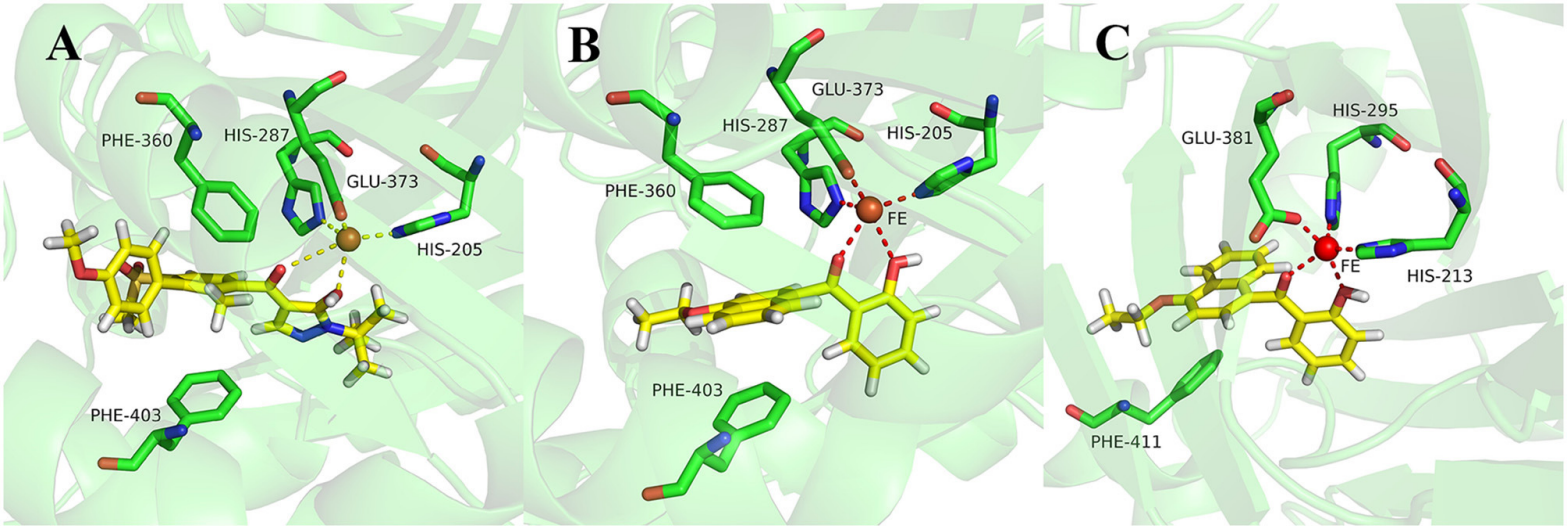
**(A)** Binding mode of DAS869 with 1TFZ. **(B)** Binding mode of compound **3h** with 1TFZ. **(C)** Binding mode of compound **3h** with Q9ARF9. Ligands DAS869 and compound **3h** were shown in yellow.

During more than 30 years of investigation, knowledge about HPPD has advanced with technology, including X-ray crystallography and bioinformatics. Consequently, 40 HPPD structures have been determined, uploaded, and stored in PDBs (Ndikuryayo et al., 2017). In order to explain the interaction of the novel compounds with HPPD at the molecular level, the docking binding modes was compared with the reported crystal structure of *At*HPPD in complex with pyrazoles inhibitor DAS869 (Pauly et al., 2013). As shown in Figure 4, both compounds **3h** and DAS869 are well-matched to the active pocket of HPPD. The simulated complex of DAS869 and inhibitors **3h** with the binding site of *At*HPPD are displayed in Figures 5A,B. The inhibitor **3h** was noticed to constitute a bidentate combination with the Fe^2+^, forming twisted square-pyramidal complex with a mainly five-coordinate. The naphthalene ring was “sandwiched” by the phenyl rings of Phe 360 and Phe 403, forming π-π interplay with the two amino acid residues, which was similar to DAS869 in complex with *At*HPPD. Compared the distances of π-π interplay, it was observed that the distance of the naphthalene ring of inhibitor **3h** with Phe 360 was 4.0 Å and that with Phe 403 was 3.4 Å; the distance of the benzene ring of DAS869 with Phe 360 was 4.5 Å and that with Phe 403 was 4.5 Å. The short distances of the naphthalene ring from Phe 360 and Phe 403 may be liable for the better *At*HPPD inhibitory activity of inhibitor **3h**.

So as to further explain the specificity interaction of the novel compounds with HPPD of others plants, six plants HPPD structures in PDB were selected for molecular docking including maize (PDB ID 1SP8), lectranthus scutellarioides (PDB ID Q9ARF9), camelina sativa (PDB ID 5YCK), brassica napus (PDB ID Q60Y65), daucus carota (PDB ID 3VLB) and hordeum vulgare (PDB ID 3TCM). The docking results showed that compound **3h** could only bind to Q9ARF9, however, the naphthalene ring didn't form a favorable sandwich π-π interaction with the key residues of Q9ARF9 (Figure 5C). These consequences illustrated that compound **3h** was safe for crops.

## Conclusion

In summary, a series of new aryl-naphthyl methanone derivatives were designed and synthesized as effective HPPD inhibitors. The bioassay indicated that the target compounds showed excellent HPPD inhibitory activity. It is inspiring that compound **3h** exhibits the best activity even better than the commercial herbicide pyrazoxyfen. This present work indicates that aryl-naphthyl methanone scaffold can be used as a potential lead structure to develop new HPPD inhibitors.

## Author Contributions

YF and FY constructed the workflow. KW and J-XK synthesized and purified the compounds. PW provided the chemicals for all biological assays and helped in relevant literature. SG and L-XZ performed the mass spectrometric analysis. YF completed the paper.

### Conflict of Interest Statement

The authors declare that the research was conducted in the absence of any commercial or financial relationships that could be construed as a potential conflict of interest.
