# Supplementary material for: Design, Synthesis, and Herbicidal Activity Evaluation of Novel Aryl-Naphthyl Methanone Derivatives
Source: Front Chem. 2019 Jan 22;7:2. doi: 10.3389/fchem.2019.00002 (PMC6349756; doi:10.3389/fchem.2019.00002)
Supplement: Supplementary file 1 [file Data_Sheet_1.docx]

Supplementary Material

**Design, Synthesis and Herbicidal Activity Evaluation of Novel Aryl-Naphthyl Methanone Derivatives**

***Ying Fu****^1^****, Kui Wang****^1^****, Peng Wang****^1^****, Jing-Xin Kang****^1^****, Shuang Gao****^1^****, Li-Xia Zhao****^1^****, Fei Ye****^1^********

^1^Department of Applied Chemistry, College of Science, Northeast Agricultural University, Harbin 150030, China

**Correspondence:**

Professor Fei Ye, Department of Applied Chemistry, College of Science, Northeast Agricultural University, Harbin 150030, China

Tel: 86-451-55191507

Email address: [yefei@neau.edu.cn](mailto:yefei@neau.edu.cn)

## Supplementary Information

Copies of detailed bioassay procedures of barnyard grass and computational work

Bioassay method:

Barnyard grass seeds were soaked in water, afterwards, the seeds were germinated in dishes in a growing chamber for 24 h. After, the seeds were sown in paper cups (10 cm × 15 cm), nine seeds per cup, soil added with a depth of 13 cm, barnyard grass seeds were incubated in a growth chamber with a 12:12 h photoperiod, 25.0 ± 1.5 °C, and 80% relative humidity. The spraying treatment was conducted at the dosage of 0.75 mmol/m^2^ when the barnyard grass achieved the two-leaf period. After 7d, the chlorophyll content was surveyed and evaluated. Each treatment was replicated three times in a completely randomized design.

Computational work:

Docking simulation was carried out utilizing CDOCKER module of the DS v2.5 to explore the binding mode of compounds. The *At*HPPD crystal structure (PDB ID: 1TFZ) was downloaded from the Protein Data Bank. The protein was prepared by removing the water, adding hydrogen and correcting the incomplete residues using “Clean Protein” tool in “Prepare Protein” module, then the protein were assigned potentials with CHARMm force field. The active site of protein was predicted and identified using “Edit binding site” module of DS v2.5 according to the native ligand and the radius was set to 10Å. The obtained receptor was used as the “Input Receptor” molecule parameter. All hit compounds subjected to first filtering processes were chosen as “Input Ligand” and docked into the active site of HPPD. The “Pose Cluster Radius” was defined as 0.5 Å for increasing the diversity of the docked poses. The Top Hits was set to 10, which means top the 10 conformations were saved for each ligand based on scoring and ranking by the negative value of CDOCKER energy. The remaining parameters were default. The best binding modes were determined by docking scores and also the comparison with available complex crystal structure of DAS869 with *At*HPPD as reference.

Copies of detailed analytical IR,^1^H NMR, ^13^C NMR, and HRMS spectra of intermediate and compounds **3a-w**

**2a:**

**1-Methoxynaphthalene**

**IR**

**^1^H NMR**


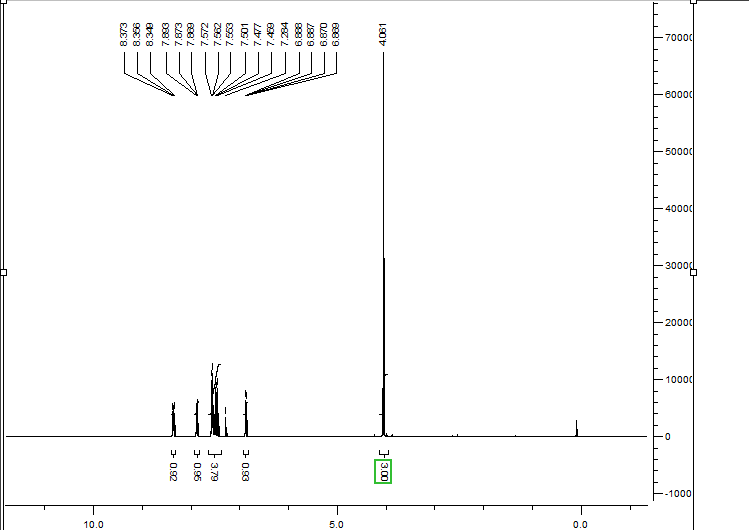


**^13^C NMR**


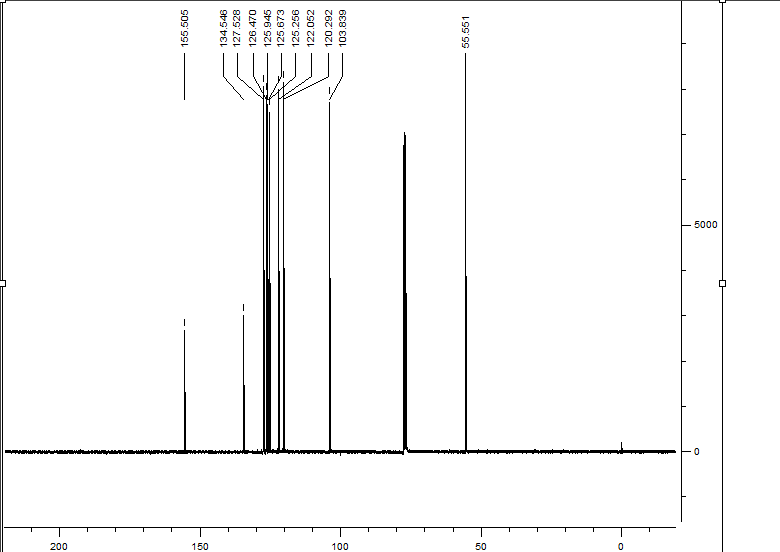


**1-Ethoxynaphthalene**

**IR**

**^1^H NMR**


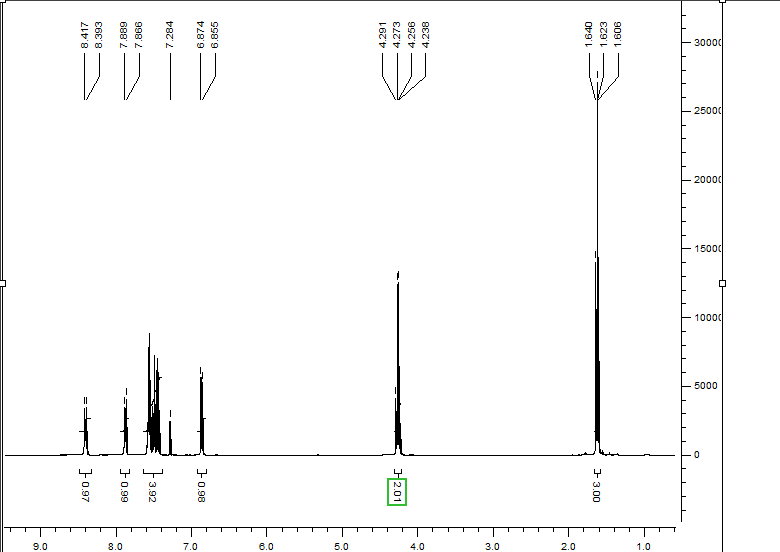


**^13^C NMR**


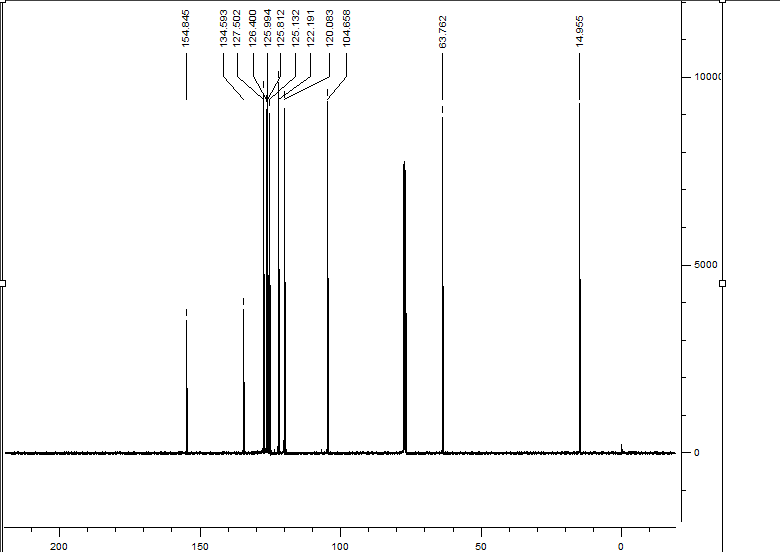


**2b:**

**2-Methoxynaphthalene**

**IR**

**^1^H NMR**

**
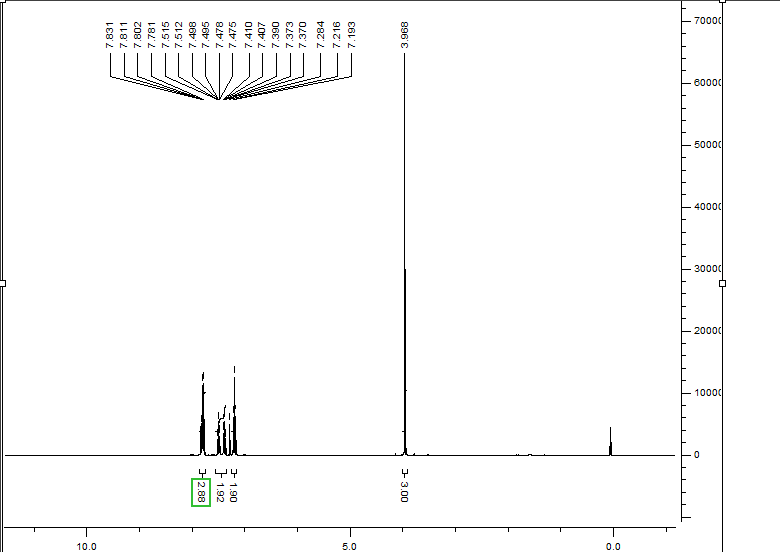
**

**^13^C NMR**

**
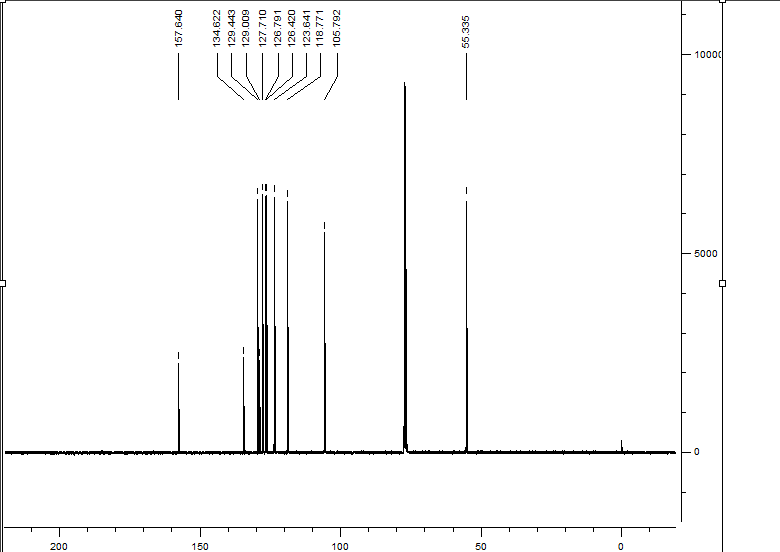
**

**(4-Ethoxy-1-naphthyl)phenyl methanone (3a)**

**IR**

**^1^H NMR**

**
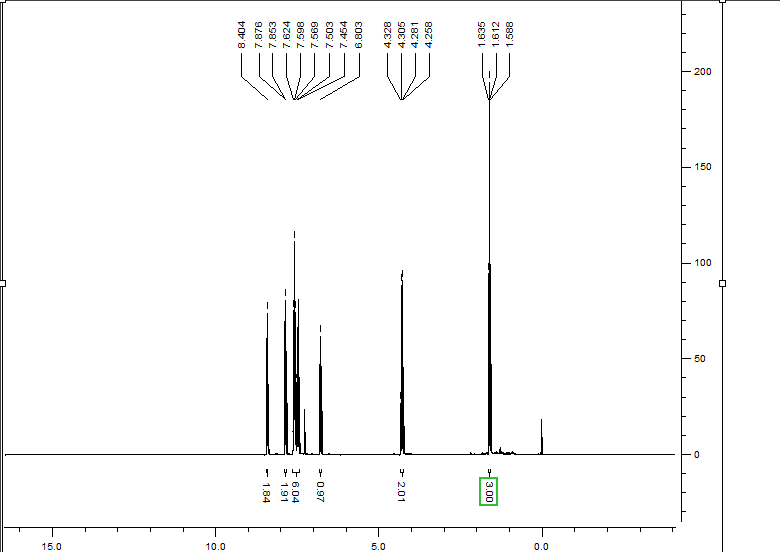
**

**^13^C NMR**

**
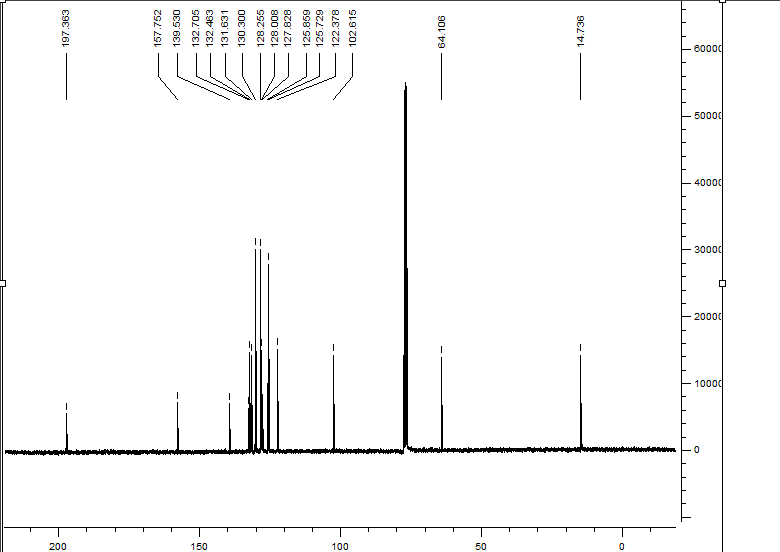
**

**HRMS**

**(4-Ethoxy-1-naphthyl)-2-chlorophenyl methanone (3b)**

**IR**

**^1^H NMR**

**
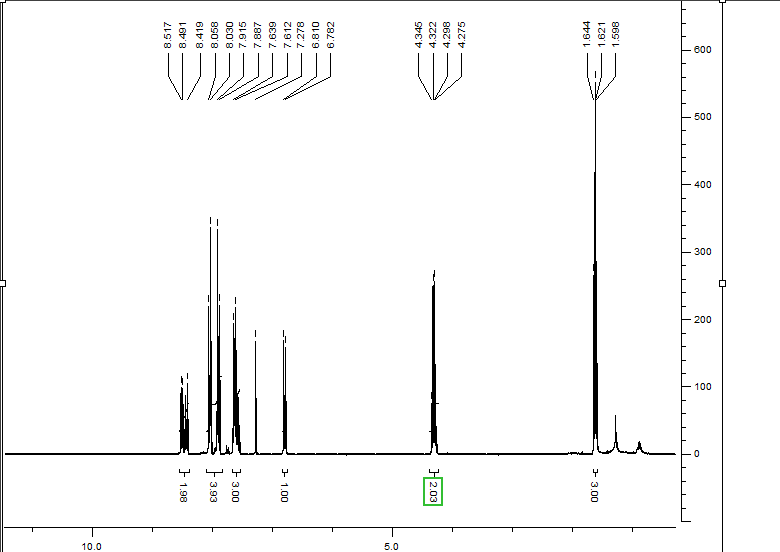
**

**^13^C NMR**

**
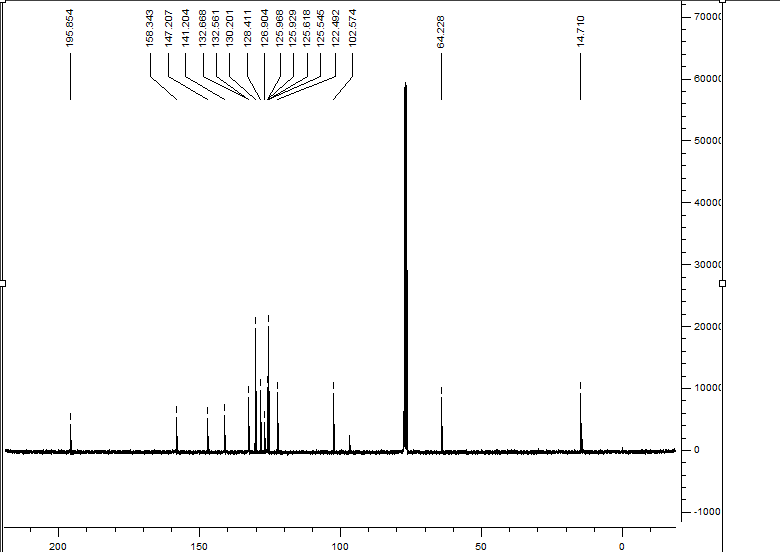
**

**HRMS**

**(4-Ethoxy-1-naphthyl)-4-chlorophenyl methanone (3c)**

**IR**

**^1^H NMR**

**
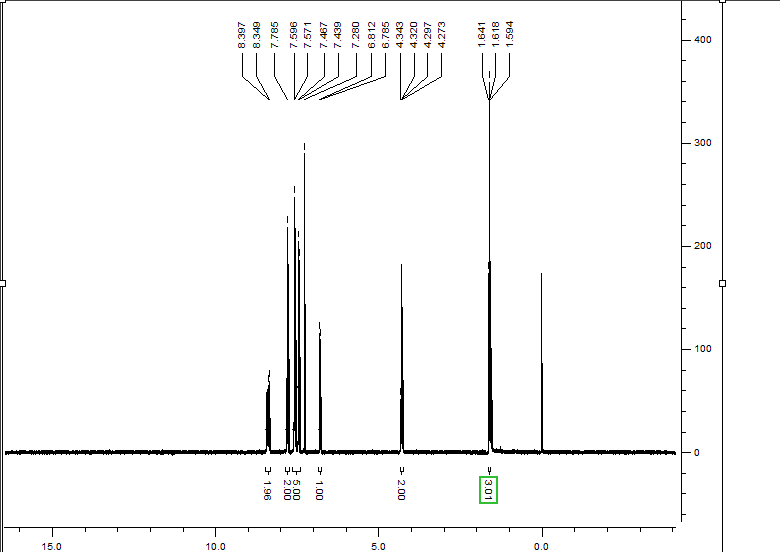
**

**^13^C NMR**

**
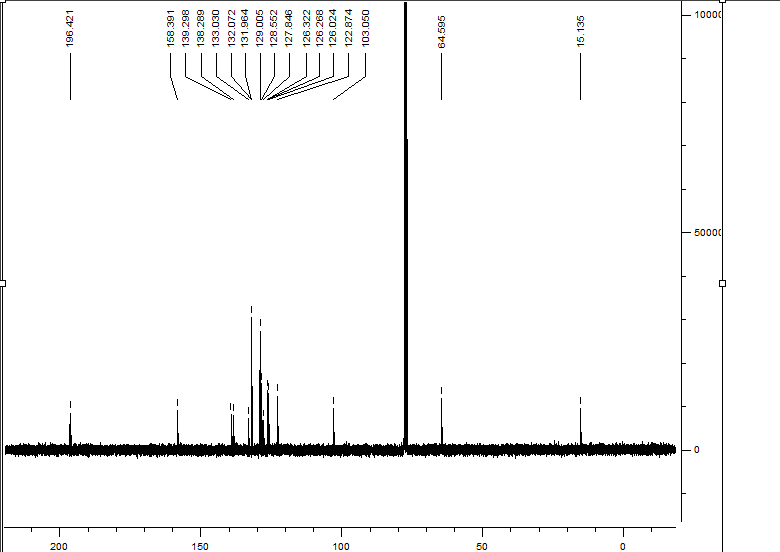
**

**HRMS**

**(4-Ethoxy-1-naphthyl)-4-fluorophenyl methanone (3d)**

**IR**

**^1^H NMR**

**
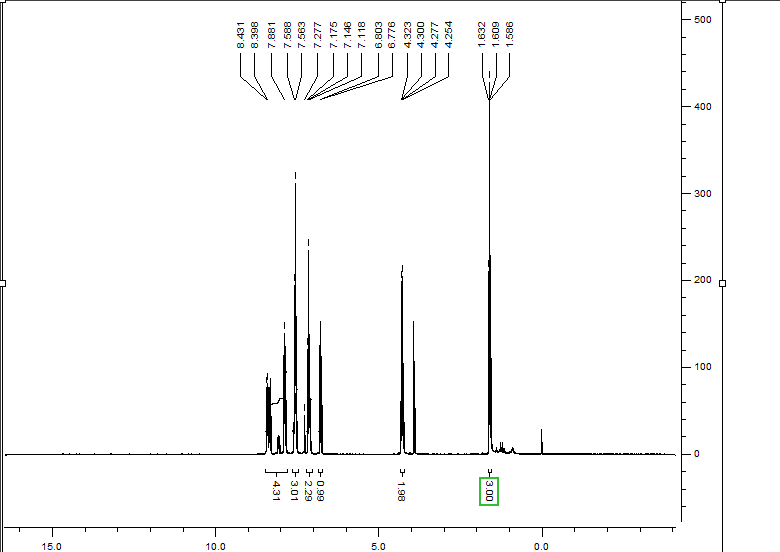
**

**^13^C NMR**

**
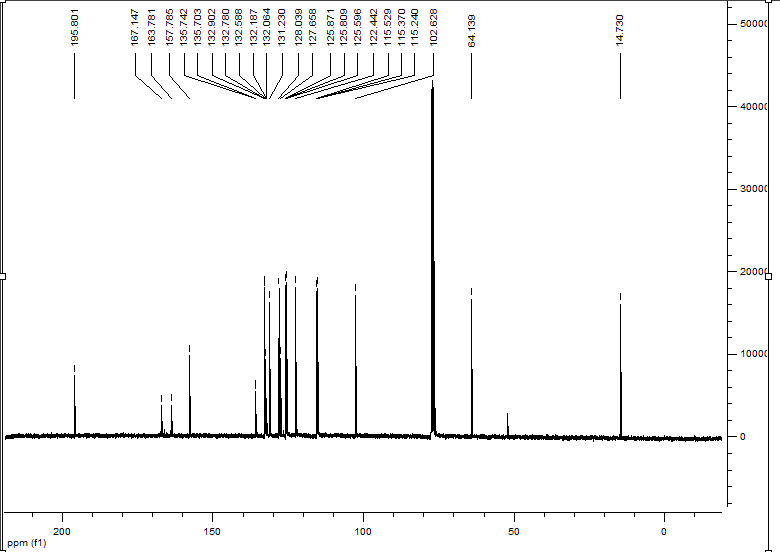
**

**HRMS**

**(4-Ethoxy-1-naphthyl)-4-trifluoromethylphenyl methanone (3e)**

**IR**

**^1^H NMR**

**
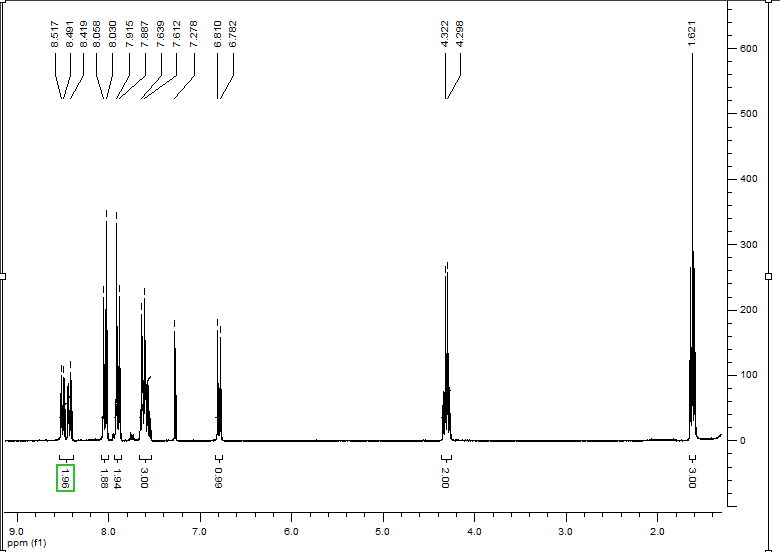
**

**^13^C NMR**

**
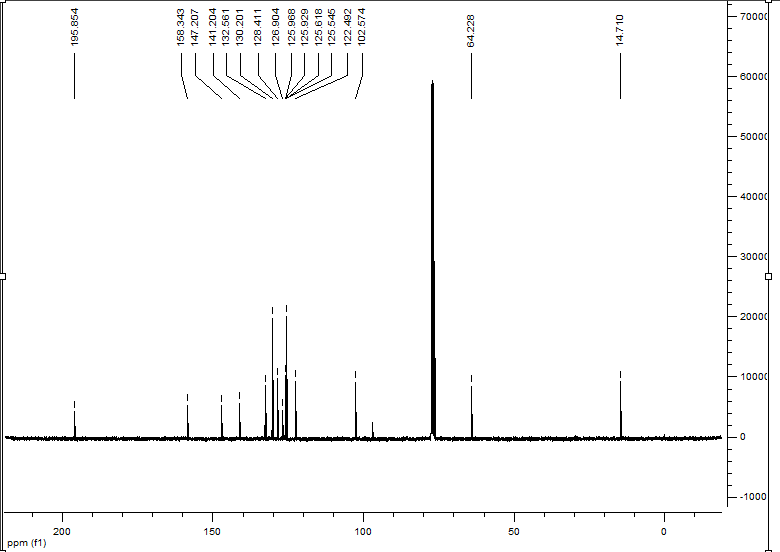
**

**HRMS**

**(4-Ethoxy-1-naphthyl)-4-nitrophenyl methanone (3f)**

**IR**

**^1^H NMR**

**
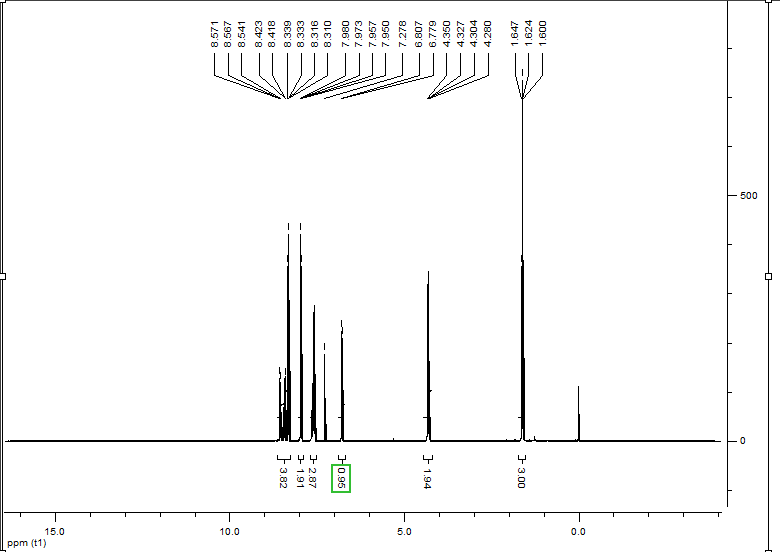
**

**^13^C NMR**

**
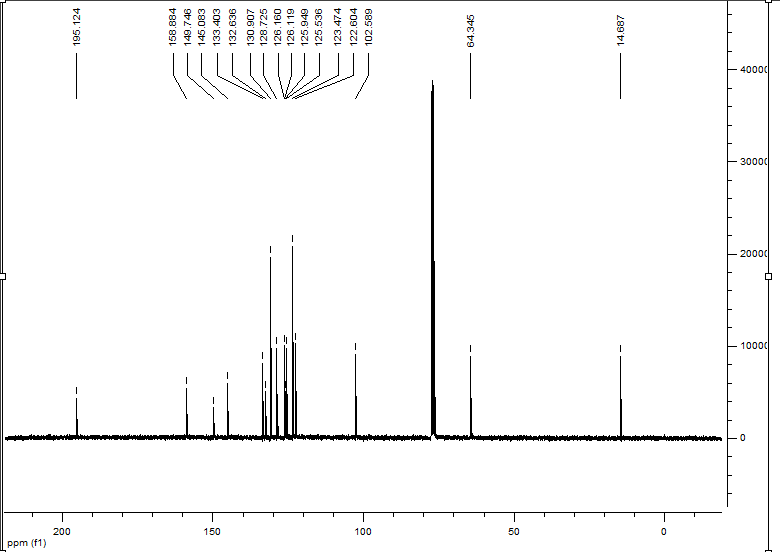
**

**HRMS**

**(4-Ethoxy-1-naphthyl)-2-methylphenyl methanone (3g)**

**IR**

**^1^H NMR**

**
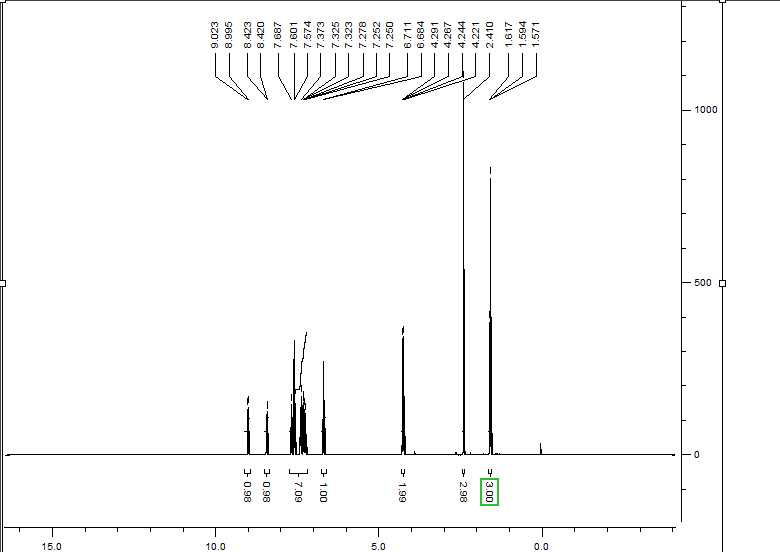
**

**^13^C NMR**

**
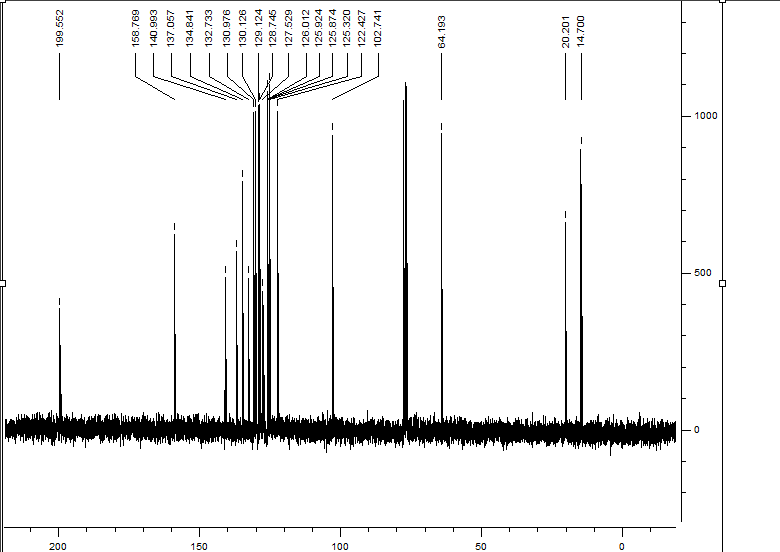
**

**HRMS**

**(4-Ethoxy-1-naphthyl)-2-hydroxyphenyl methanone (3h)**

IR

**^1^H NMR**

**
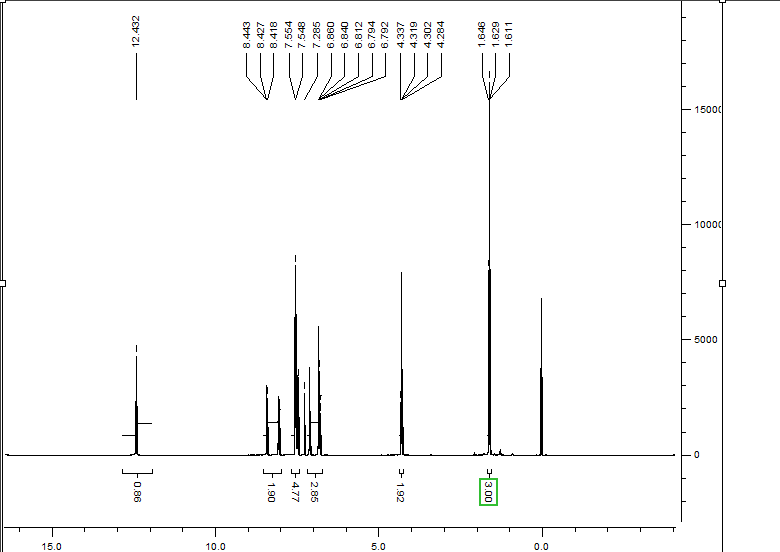
**

**^13^C NMR**

**
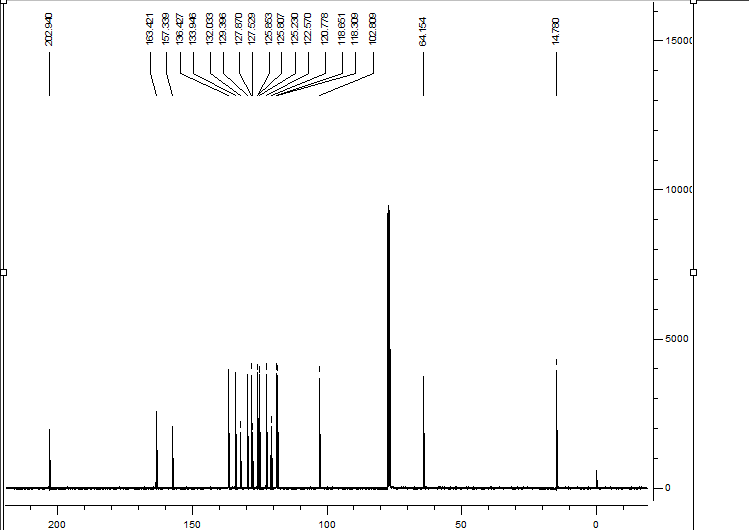
**

**HRMS**

**(4-Ethoxy-1-naphthyl)-2-ethoxyphenyl methanone (3i)**

**IR**

**^1^H NMR**

**
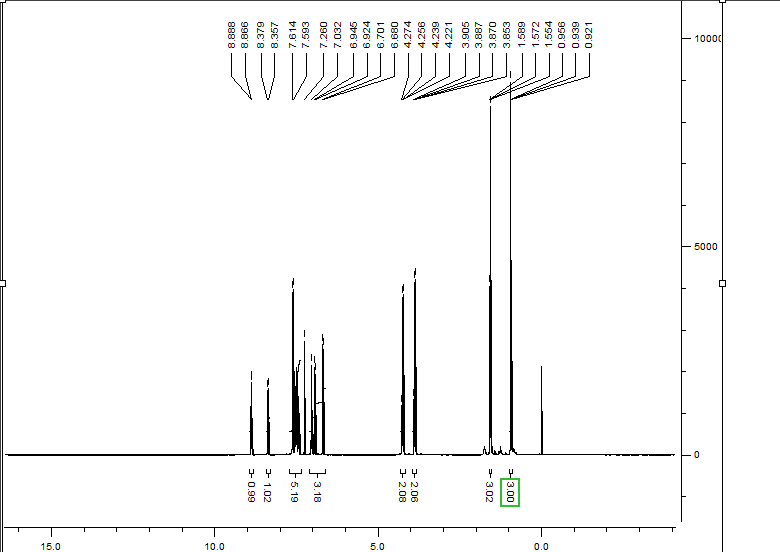
**

**^13^C NMR**

**
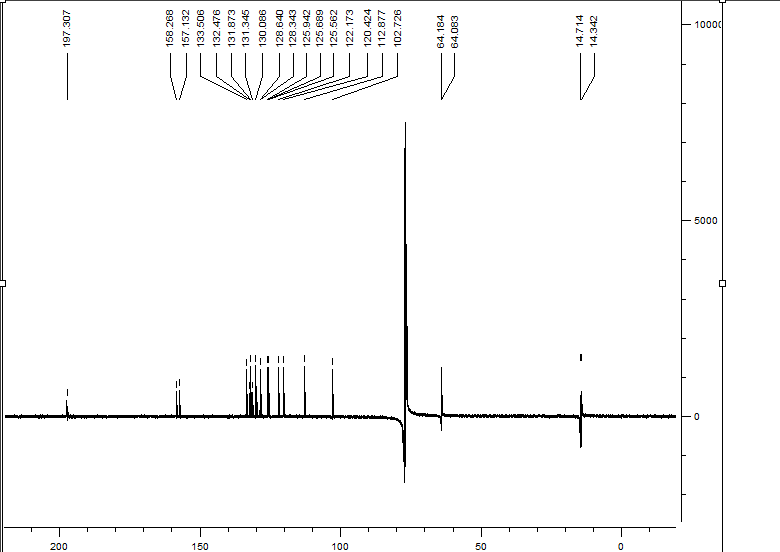
**

**HRMS**

**(4-Methoxy-1-naphthyl)-2-hydroxyphenyl methanone (3j)**

**IR**

**^1^H NMR**

**
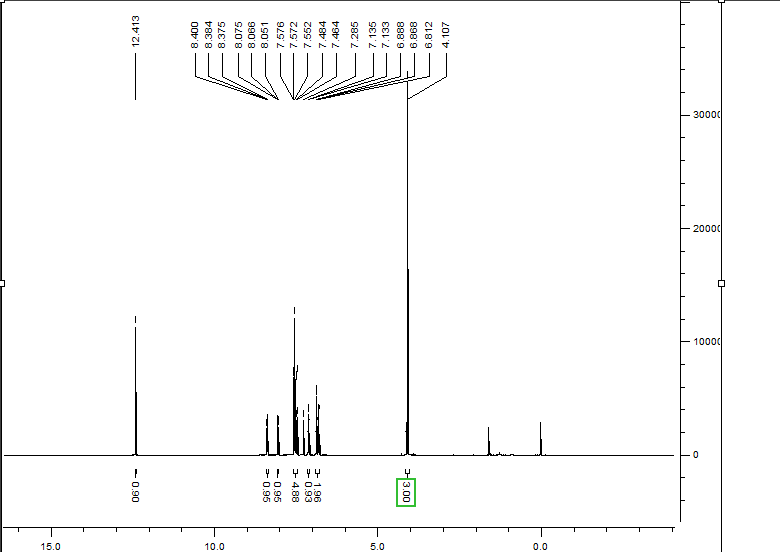
**

**^13^C NMR**

**
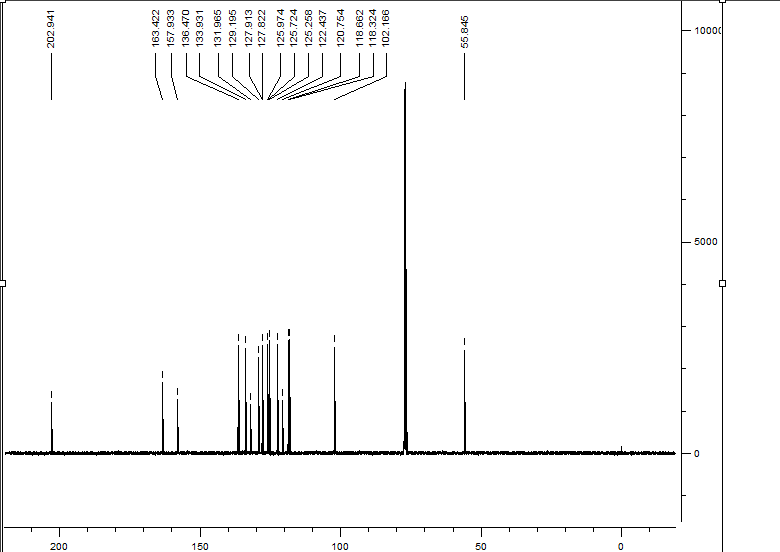
**

**HRMS**

**(4-Methoxy-1-naphthyl)-4-methylphenyl methanone (3k)**

**IR**

**^1^H NMR**

**
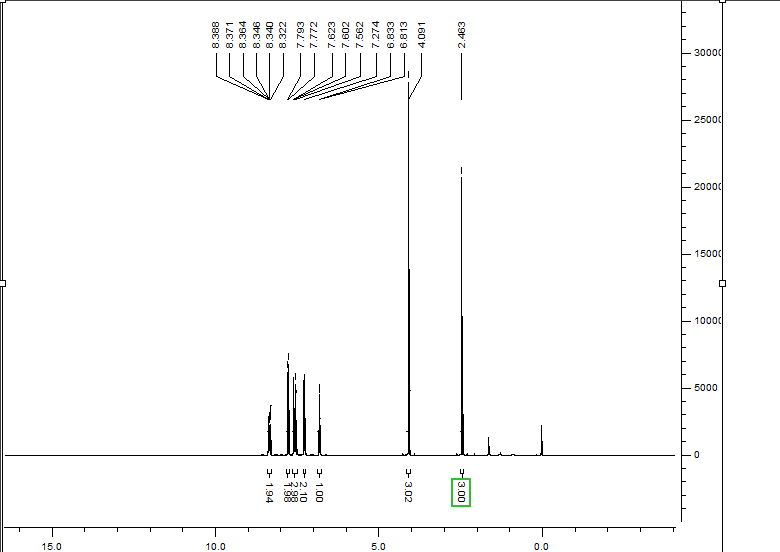
**

**^13^C NMR**

**
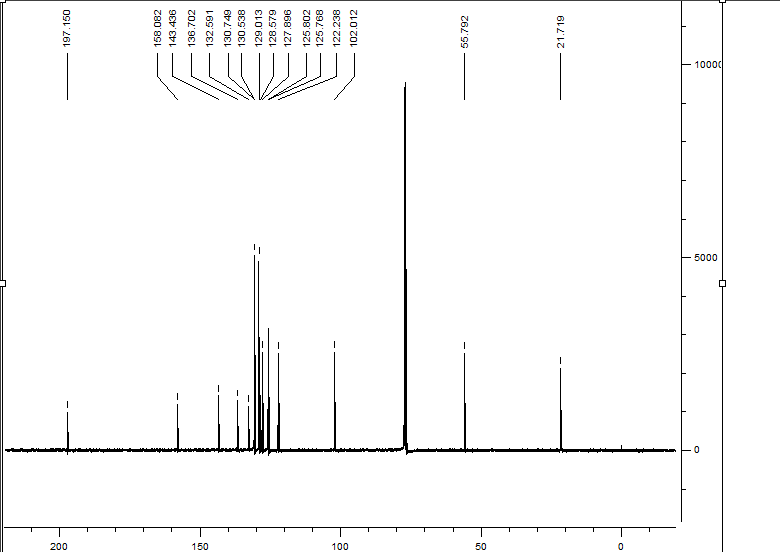
**

**HRMS**

**(4-Methoxy-1-naphthyl)-4-nitrophenyl methanone (3l)**

**IR**

**^1^H NMR**

**
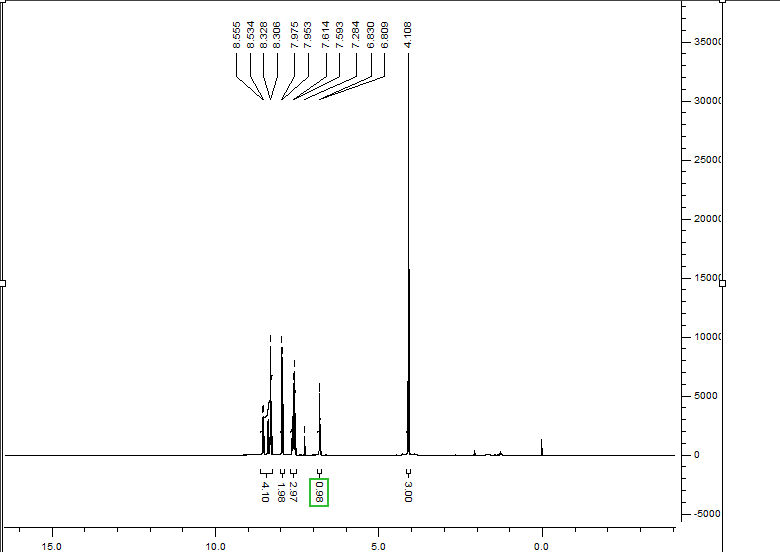
**

**^13^C NMR**

**
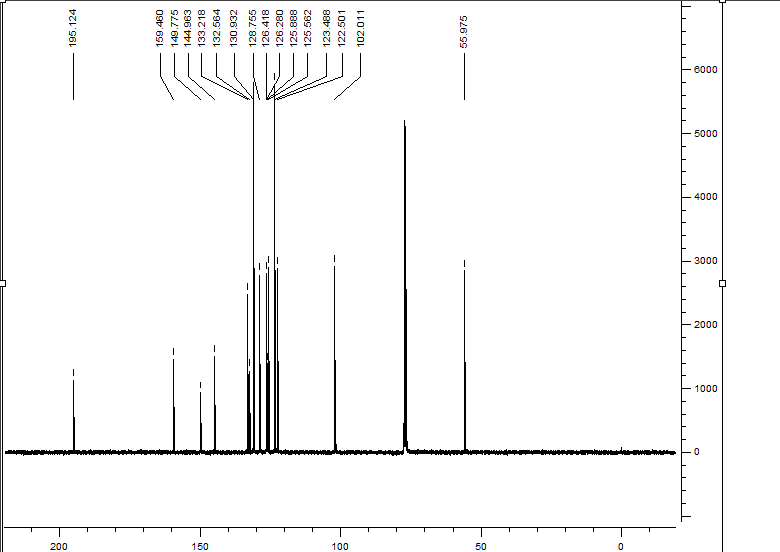
**

**HRMS**

**(2-Methoxy-1-naphthyl)-4-trifluoromethylphenyl methanone (3m)**

**IR**

**^1^H NMR**

**
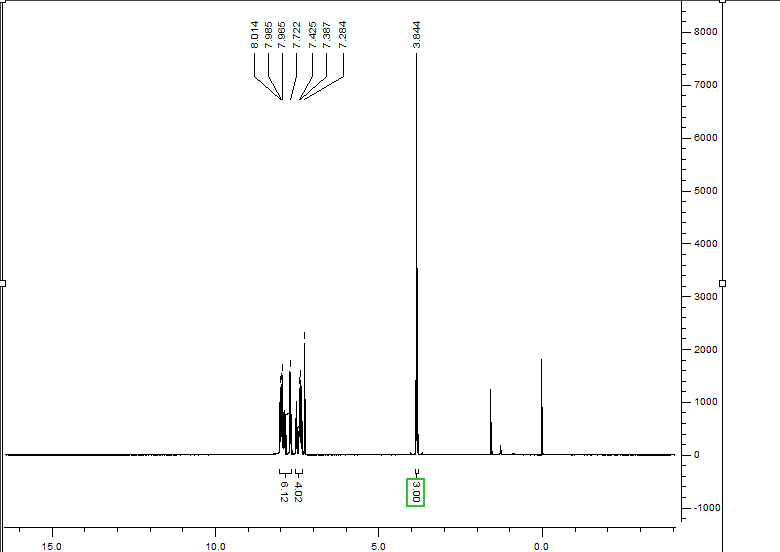
**

**^13^C NMR**

**
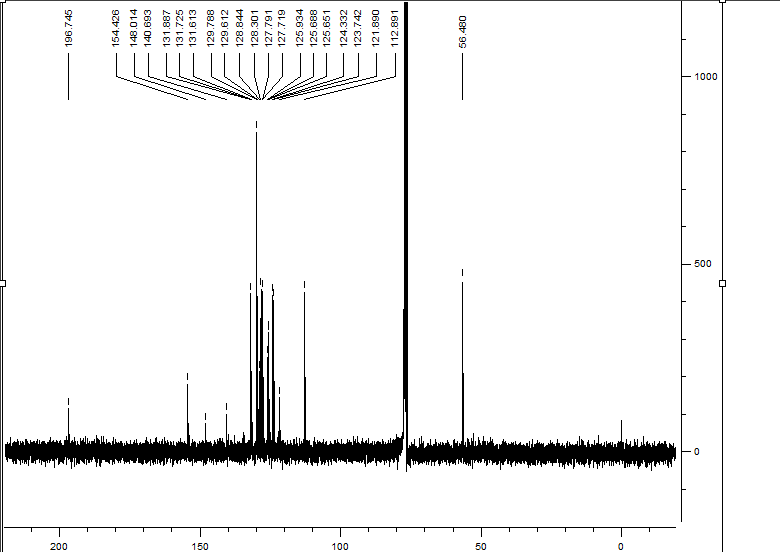
**

**HRMS**

**(2-Methoxy-1-naphthyl)-4-nitrophenyl methanone (3n)**

**IR**

**^1^H NMR**

**
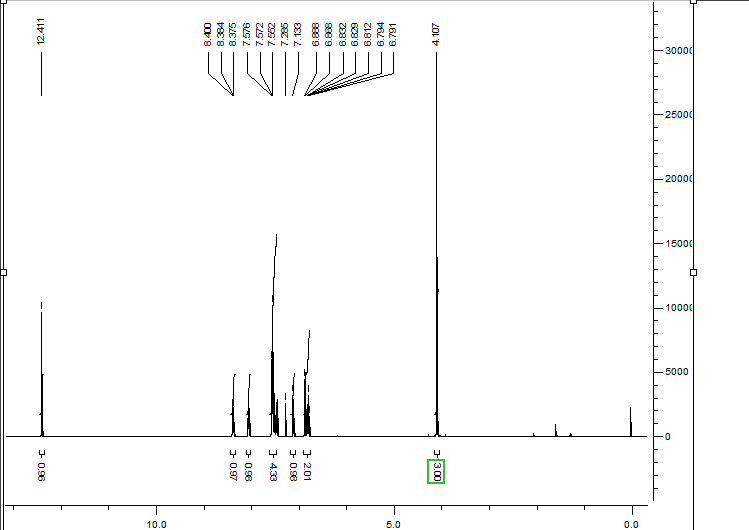
**

**^13^C NMR**

**
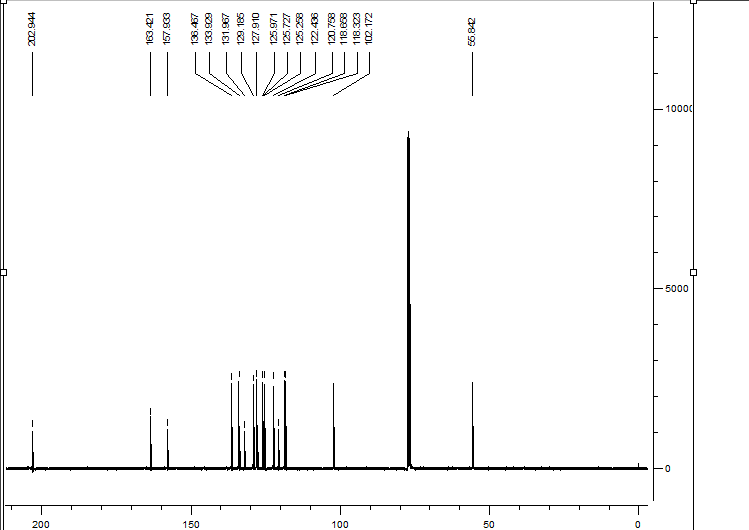
**

**HRMS**

**4-(1-Methyl-3-trifluoromethyl)pyrazole-(4-ethoxy-1-naphthyl) methanone (3o)**

**IR**

**^1^H NMR**

**
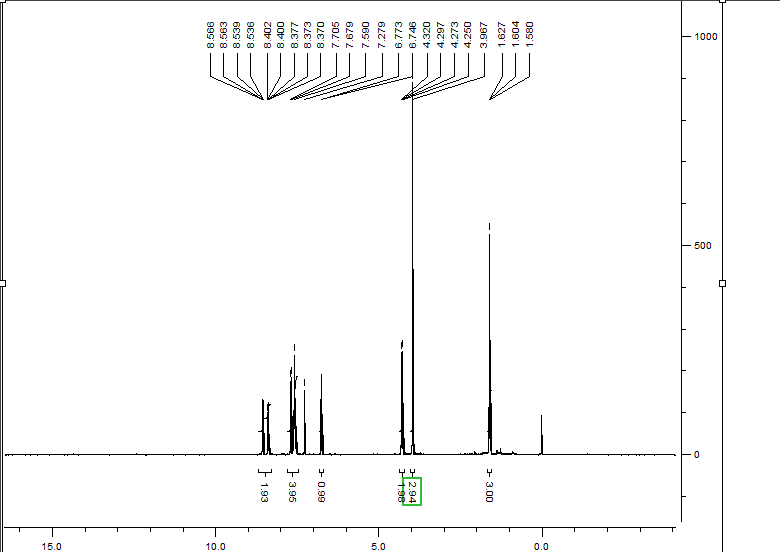
**

**^13^C NMR**

**
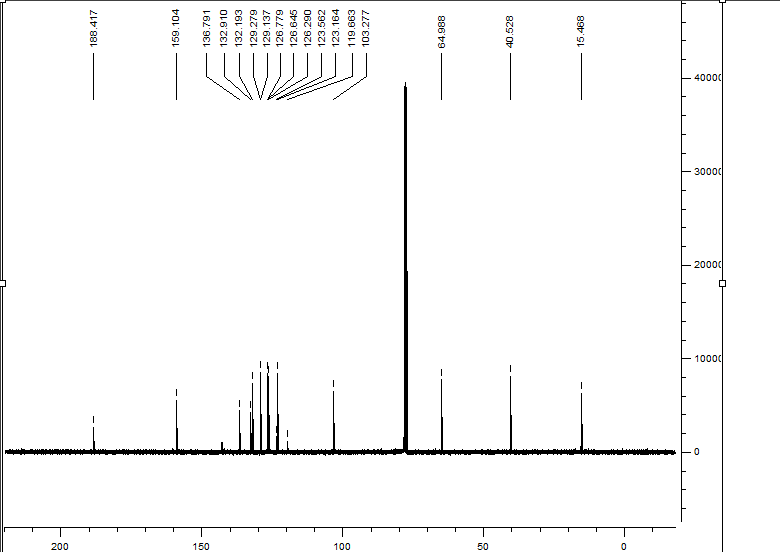
**

**HRMS**

**1-(2,4-Dichlorophenyl)-5-(trichloromethyl)-1H-1,2,4-triazole-(4-ethoxy-1-naphthyl) methanone (3p)**

**IR**

**^1^H NMR**

**
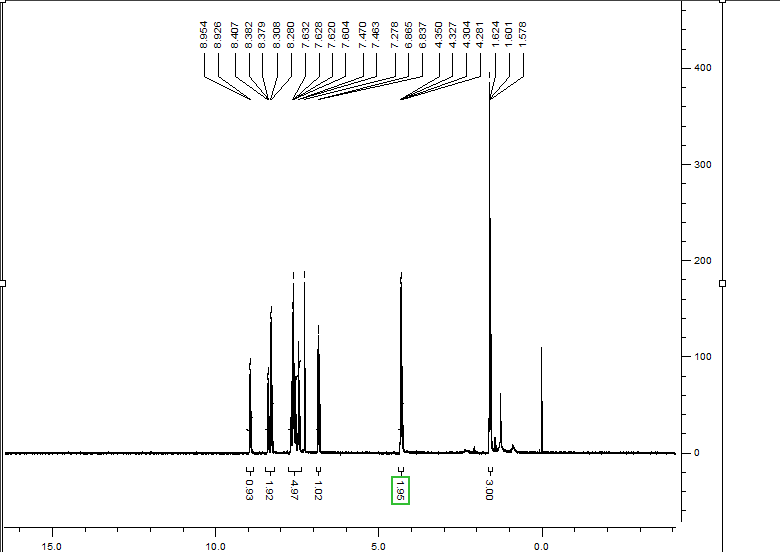
**

**^13^C NMR**

**
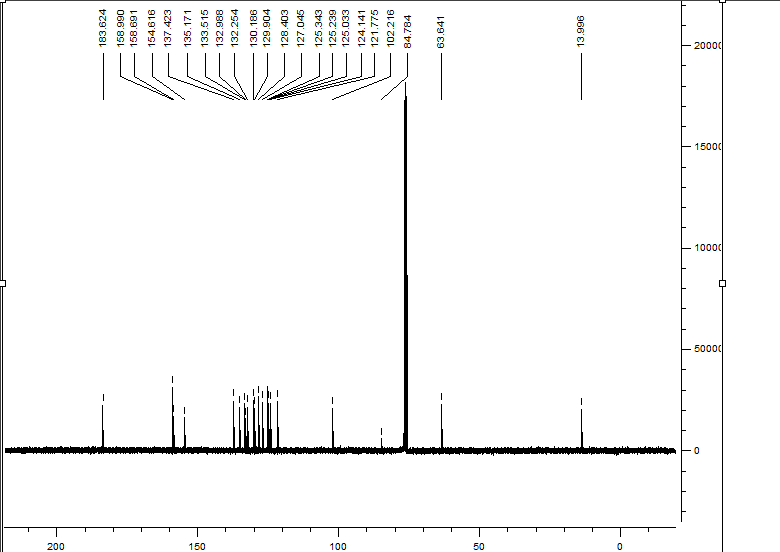
**

**HRMS**

**5-Methyl-3-phenyl-4-isoxazole-(4-ethoxy-1-naphthyl) methanone (3q)**

**IR**

**^1^H NMR**

**
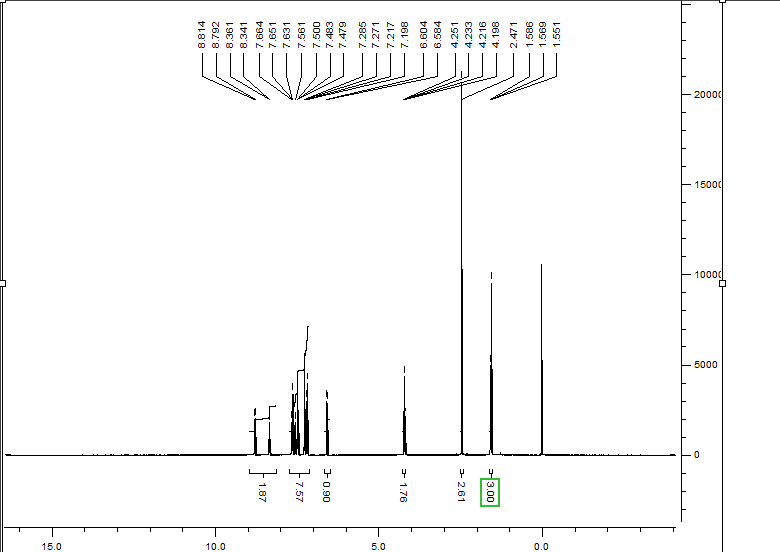
**

**^13^C NMR**

**
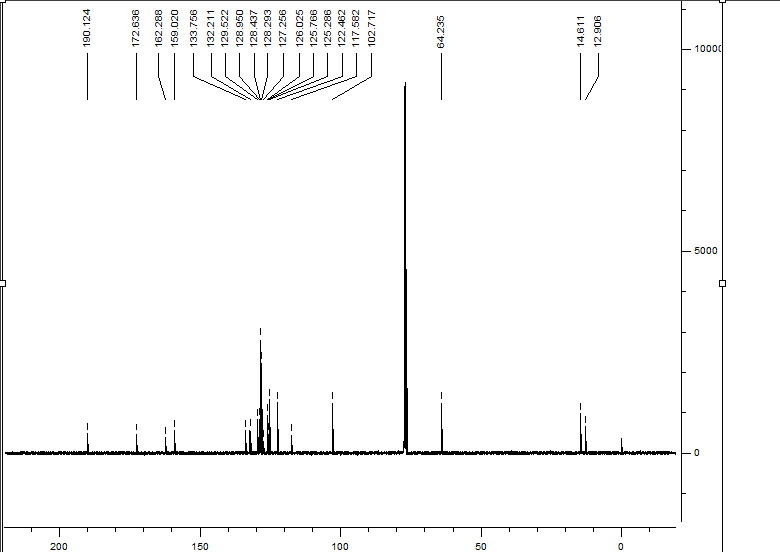
**

**HRMS**

**5-Methyl-3-(2-fluoro-6-chlorophenyl)-4-isoxazole-(1-ethoxy-4-naphthyl) methanone (3r)**

**IR**

**^1^H NMR**

**
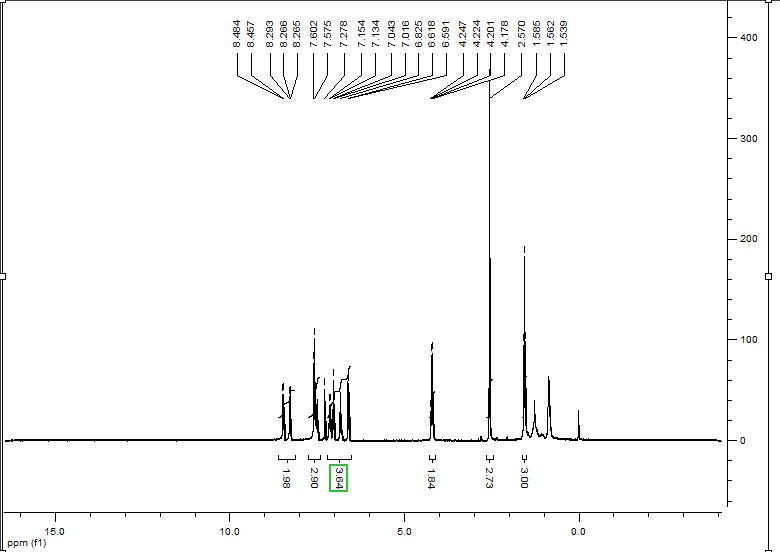
**

**^13^C NMR**

**
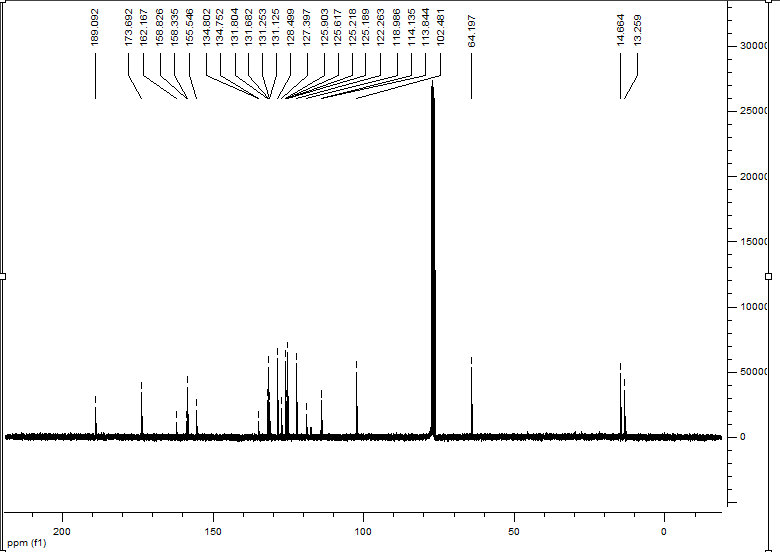
**

**HRMS**

**5-Methyl-3-(2-fluoro-6-chlorophenyl)-4-isoxazole-(1-methoxy-4-naphthyl) methanone (3s)**

**IR**

**^1^H NMR**

**
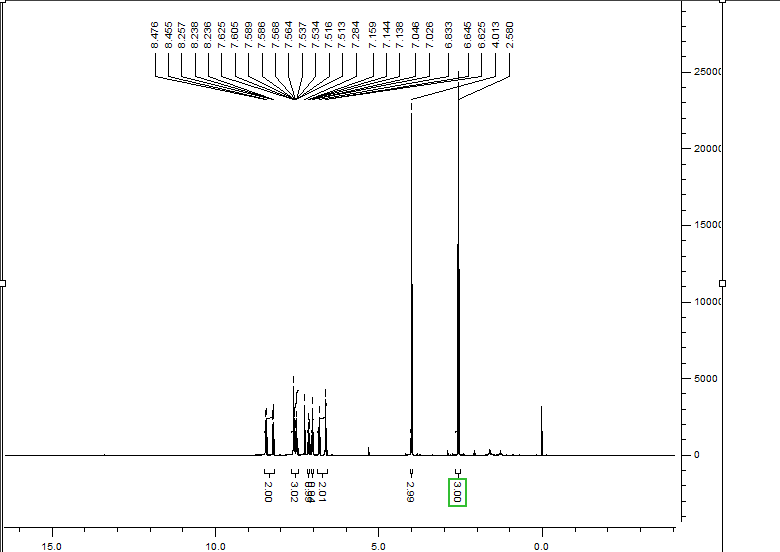
**

**^13^C NMR**

**
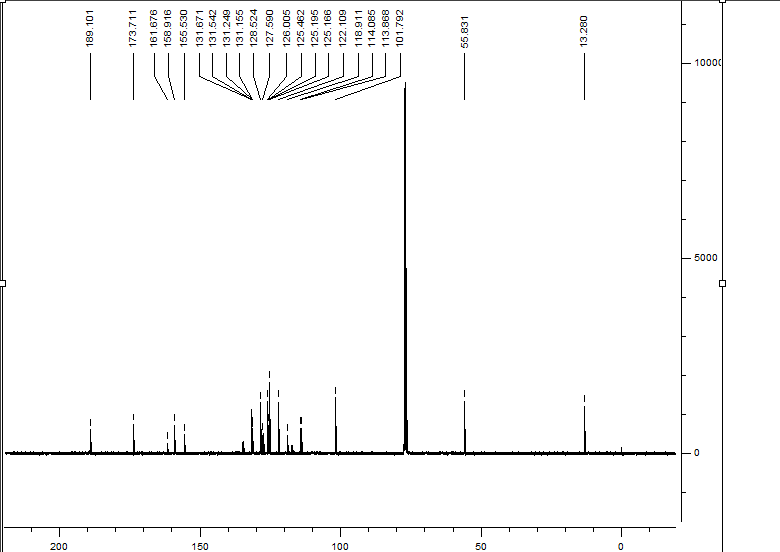
**

**HRMS**

**5-Methyl-3-phenyl-4-isoxazole-(4-ethoxy-1-naphthyl) methanone (3t)**

**^1^H NMR**

**
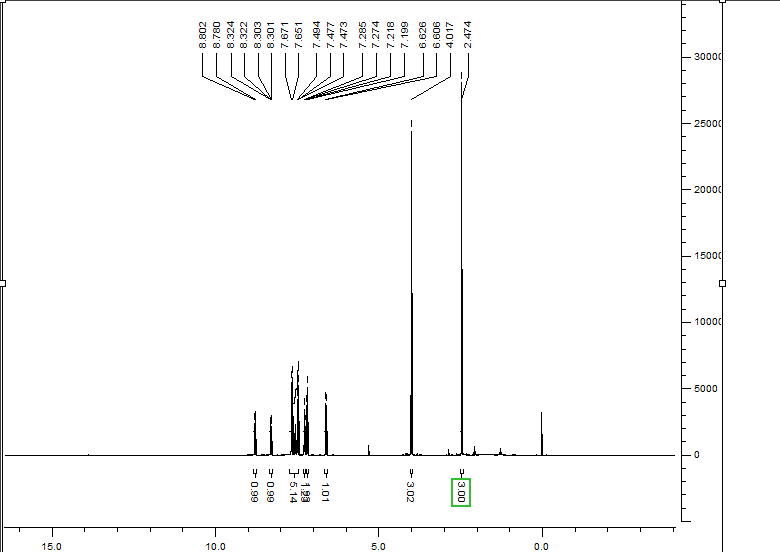
**

**^13^C NMR**

**
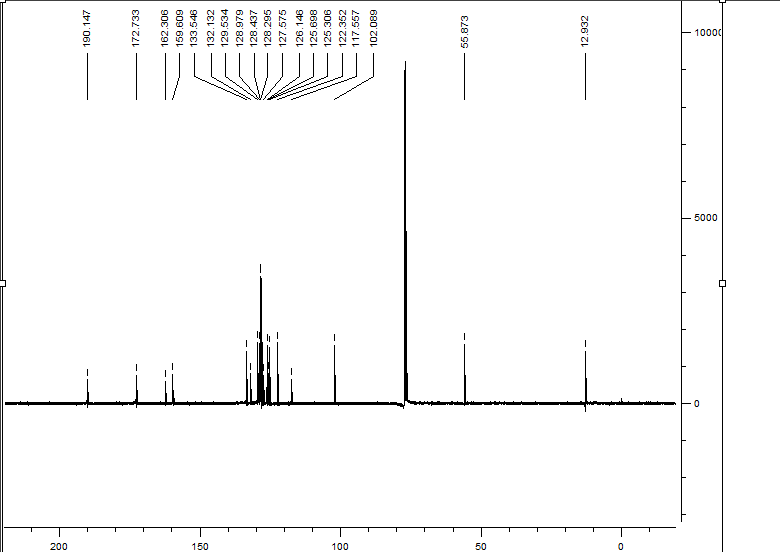
**

**HRMS**

**(4-Ethoxy-1-naphthyl)furanone (3u)**

**IR**

**^1^H NMR**

**
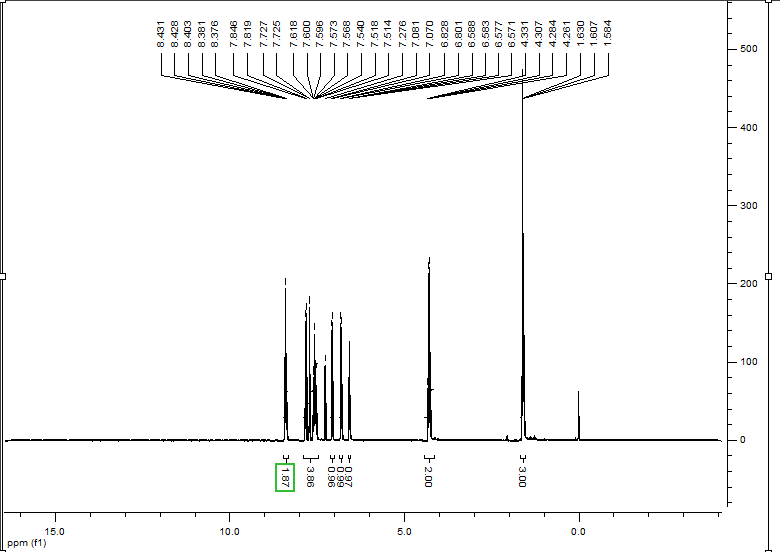
**

**^13^C NMR**

**
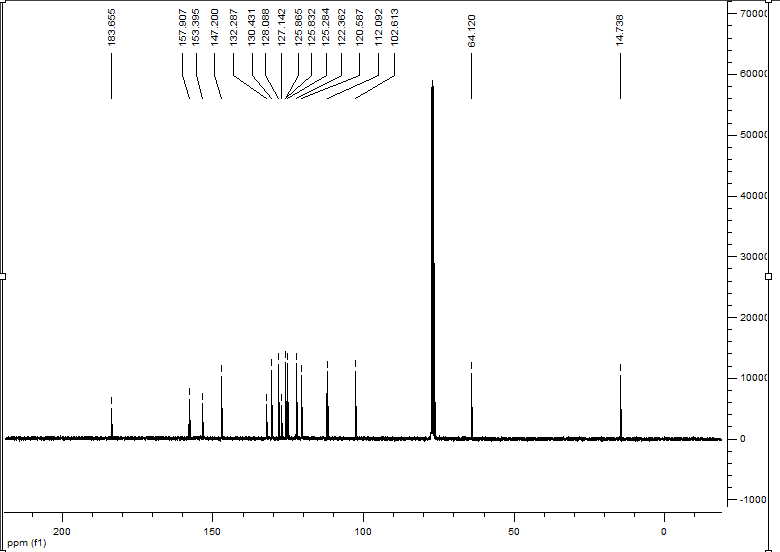
**

**HRMS**

**(4-Methoxy-1-naphthyl)furanone (3v)**

**IR**

**^1^H NMR**

**
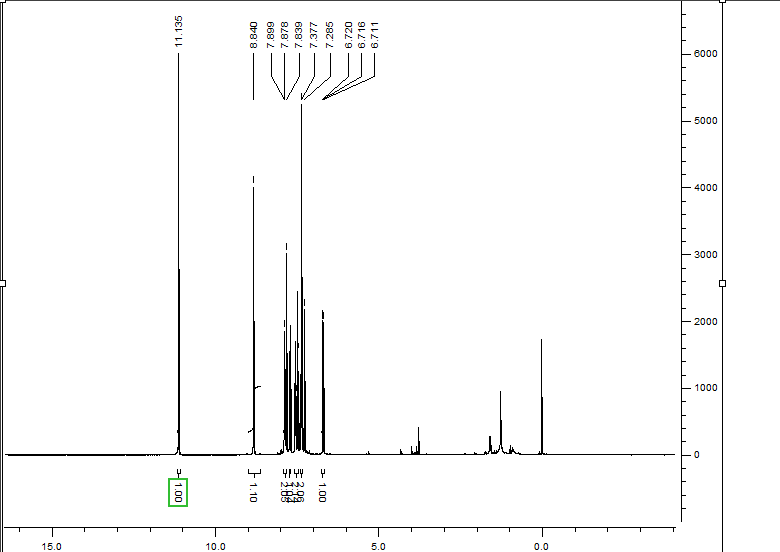
**

**^13^C NMR**

**
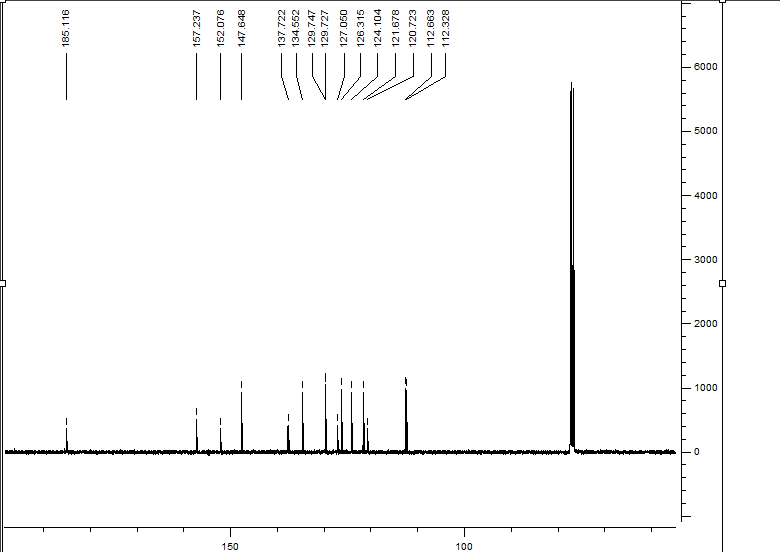
**

**HRMS**

**(2-Hydroxyl-1-naphthyl)furanone (3w)**

**IR**

**^1^H NMR**

**
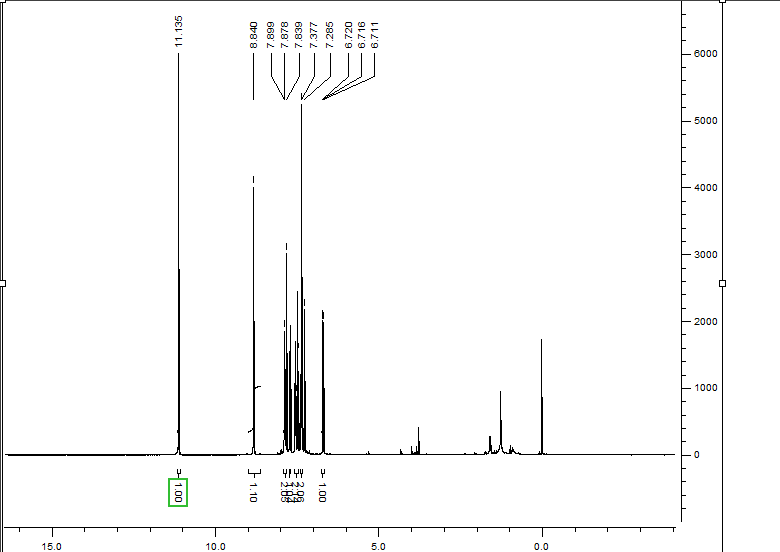
**

**^13^C NMR**

**
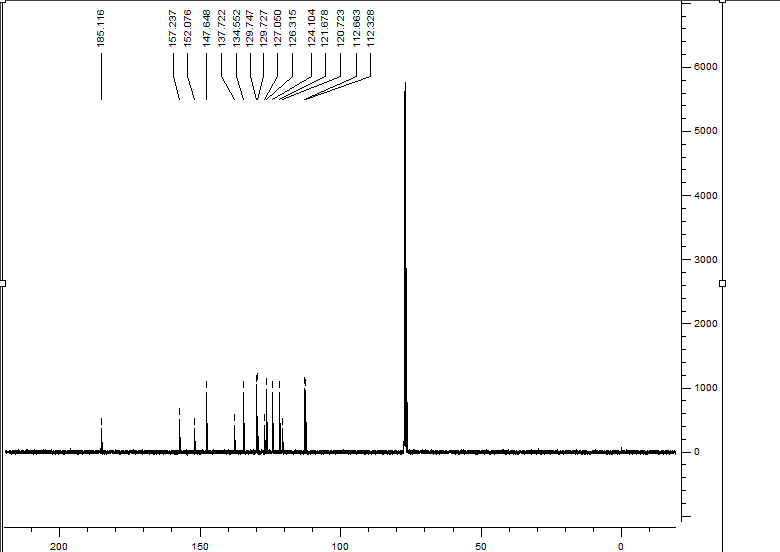
**

**HRMS**
